# A non-canonical RNA degradation pathway suppresses RNAi-dependent epimutations in the human fungal pathogen *Mucor circinelloides*

**DOI:** 10.1371/journal.pgen.1006686

**Published:** 2017-03-24

**Authors:** Silvia Calo, Francisco E. Nicolás, Soo Chan Lee, Ana Vila, Maria Cervantes, Santiago Torres-Martinez, Rosa M. Ruiz-Vazquez, Maria E. Cardenas, Joseph Heitman

**Affiliations:** 1 Department of Molecular Genetics and Microbiology, Duke University Medical Center, Durham, North Carolina, United States of America; 2 Department of Genetics and Microbiology, Faculty of Biology, University of Murcia, Murcia, Spain; Stanford University School of Medicine, UNITED STATES

## Abstract

Mucorales are a group of basal fungi that includes the casual agents of the human emerging disease mucormycosis. Recent studies revealed that these pathogens activate an RNAi-based pathway to rapidly generate drug-resistant epimutant strains when exposed to stressful compounds such as the antifungal drug FK506. To elucidate the molecular mechanism of this epimutation pathway, we performed a genetic analysis in *Mucor circinelloides* that revealed an inhibitory role for the non-canonical RdRP-dependent Dicer-independent silencing pathway, which is an RNAi-based mechanism involved in mRNA degradation that was recently identified. Thus, mutations that specifically block the mRNA degradation pathway, such as those in the genes *r3b2* and *rdrp3*, enhance the production of drug resistant epimutants, similar to the phenotype previously described for mutation of the gene *rdrp1*. Our genetic analysis also revealed two new specific components of the epimutation pathway related to the quelling induced protein (qip) and a Sad-3-like helicase (*rnhA*), as mutations in these genes prevented formation of drug-resistant epimutants. Remarkably, drug-resistant epimutant production was notably increased in *M*. *circinelloides f*. *circinelloides* isolates from humans or other animal hosts. The host-pathogen interaction could be a stressful environment in which the phenotypic plasticity provided by the epimutant pathway might provide an advantage for these strains. These results evoke a model whereby balanced regulation of two different RNAi pathways is determined by the activation of the RNAi-dependent epimutant pathway under stress conditions, or its repression when the regular maintenance of the mRNA degradation pathway operates under non-stress conditions.

## Author summary

Mucormycosis is a fungal infection that is attracting the attention of both clinical and research communities because of the lack of effective antifungal treatments and its often fatal prognosis. Our previous studies revealed an RNAi-mediated epimutation mechanism that operates in the casual human fungal pathogens (Mucorales species) and which might underlie the lack of efficacy of some antifungal treatments. This epimutation mechanism represses the expression of antifungal drug target genes and thereby generates antifungal drug resistant strains. Here, we studied the regulation and identified new components of the epimutation pathway. We found that a newly identified mRNA degradation pathway, named the non-canonical RdRP-dependent Dicer-independent silencing pathway, exerts an inhibitory effect on the RNAi-mediated epimutation mechanism and operates during growth under non-stressful conditions. Interestingly, the RNAi-based epimutation mechanism is more active in *M*. *circinelloides f*. *circinelloides* isolates from human and other animal sources, suggesting that this mechanism may influence host-pathogen interactions. These results further our understanding of the mechanisms deployed by fungal pathogens to survive and adapt under stressful environmental conditions that may include the host niche.

## Introduction

RNA interference (RNAi) is a complex gene regulatory system that blocks the expression of target genes and is highly conserved in most eukaryotic organisms. The discovery of RNAi mechanisms, along with the development of next generation sequencing technologies, has revealed a myriad of regulatory small RNAs that is revolutionizing our knowledge of RNA functions. In this system, the common core of the different RNAi pathways represses gene expression through a homology-dependent mechanism mediated by small non-conding RNAs (sRNAs). These sRNAs are produced by RNaseIII Dicer enzymes from double-stranded RNA (dsRNA) precursors, and then loaded into an RNA-induced silencing complex (RISC) to function as probes to recognize the target RNA. Once captured the target RNA is either degraded or its translation is blocked by Argonaute proteins, which are the main components of the RISC complex. Besides this common core, in both fungi and plants the RNAi mechanism also requires RNA-dependent RNA polymerases (RdRPs) to synthesize dsRNA from single-stranded RNA (ssRNA) [1, 2]. RNAi was first described as a defense mechanism against invasive nucleic acids. However, diverse regulatory roles of the RNAi machinery have been documented since its discovery, including post-transcriptional (mRNA accumulation and translation) and transcriptional (chromatin silencing) gene regulation, as well as programmed DNA rearrangements and genome surveillance [3].

Quelling, meiotic silencing by unpaired DNA (MSUD), and sex induced silencing (SIS) are distinct RNAi pathways that have been described in *Neurospora crassa* and *Cryptococcus neoformans*, respectively, and new RNAi pathways are still being discovered in other fungi [4, 5]. In fact, two novel and unusual mechanisms have been recently reported in the human fungal pathogen *Mucor circinelloides* that differ from the canonical exogenously or endogenously activated RNAi pathways described previously [6, 7]. These are an RNAi-dependent epimutation pathway [4] and an RdRP-dependent Dicer-independent degradation cascade that acts on endogenous mRNA [5]. These discoveries position *M*. *circinelloides* as an innovative model to elucidate the intrinsic and complex RNAi pathways and their biological functions.

The RNAi-dependent epimutation mechanism was discovered due to the ability of *M*. *circinelloides* to develop transient resistance to the antifungal drug FK506. *Mucor* is a dimorphic fungus that typically grows as a filamentous hyphae yet only grows as a yeast under low oxygen and high CO_2_ conditions [8] or when the calcineurin pathway is inhibited or mutated [9, 10]. The antifungal drugs FK506 and rapamycin interact with the prolyl isomerase FKBP12 [11], conserved throughout eukaryotes, and the resulting protein-drug complexes inhibit the protein phosphatase calcineurin and the TOR kinase, respectively [12, 13]. Exposure to FK506 blocks hyphal growth of *M*. *circinelloides* and enforces yeast growth. However, *M*. *circinelloides* is able to acquire resistance to FK506 and return to hyphal growth via two distinct routes: the first involves Mendelian mutations of the *fkbA* gene encoding FKBP12 or the calcineurin A or B subunit genes *cnaA* and *cnbR* [10]; the second route involves an epigenetic RNAi-mediated pathway based on reversible silencing of the *fkbA* gene [4].

As mentioned above, a recently published study described a novel RdRP-dependent Dicer-independent degradation mechanism for endogenous mRNA [5]. This unusual Dicer-independent regulatory pathway controls the expression of conserved and highly expressed genes via specific degradation of the corresponding target mRNAs. A new RNase III-like protein named R3B2 has been found to function as the primary RNase protein in this pathway instead of Dicer. R3B2 is also involved in the canonical RNAi pathway, likely by functioning in combination with the Dicer proteins [5], although its specific role in this pathway is still unknown. How R3B2 discriminates between both pathways is also unknown. One possibility is that interaction with Dicer or the RdRP proteins targets the RNA for degradation via either the canonical RNAi mechanism or the Dicer-independent pathway, respectively.

Here, we identify a link between the RNAi-dependent epimutation pathway and the RdRP-dependent Dicer-independent RNA degradation pathway. Our study dissecting the role in the epimutation mechanism of different genetic elements involved in canonical and non-canonical RNAi reveals a competitive relationship between these pathways as mutants defective in the RNA degradation mechanism produced epimutants at a much higher frequency relative to the wild type. These results reveal that the epimutation pathway is either negatively controlled by the non-canonical RdRP-dependent degradation pathway, or the two compete for target mRNAs, highlighting the complexity of RNAi-related pathway interactions in fungi. Furthermore, the enhanced ability to activate the epimutation pathway exhibited by several *M*. *circinelloides f*. *circinelloides* isolates from human and animal sources that are virulent in a murine model suggests that the epimutant pathway may be relevant to the host-pathogen interaction.

## Results

### The RNaseIII-like protein R3B2 represses epimutation formation

In light of the results implicating R3B2 in RNA metabolic pathways, and to better understand the relationship between the three different known RNAi mechanisms that operate in *M*. *circinelloides*, we investigated whether R3B2 functions in the RNAi-dependent epimutation mechanism. To this end, two independent *r3b2* null mutant strains (MU412 and MU429, [5]) were incubated in the presence of 1 μg/ml FK506 on solid medium [4]. *M*. *circinelloides* grows as a yeast on YPD media supplemented with 1 μg/ml of FK506 [9], but after 6 to 15 days of incubation at 25°C, the fungus can develop resistance to the drug either via spontaneous Mendelian mutations in the target genes, *fkbA* or the calcineurin genes, or via an epigenetic RNAi-mediated pathway [4]. These FK506 resistance mechanisms restore *Mucor* growth as a filamentous fungus in a stable or unstable fashion, depending on the mechanism involved (mutation or epimutation) [4].

**Fig 1:**
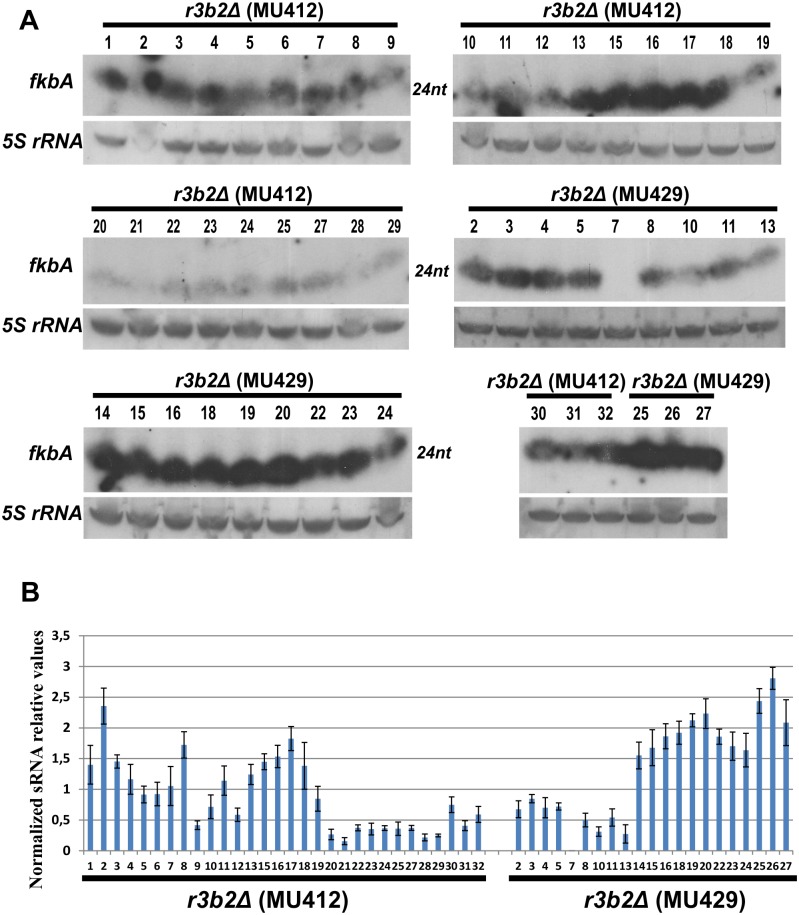
*fkbA* sRNAs are present in the FK506 resistant epimutants isolates of the *r3b2*Δ mutant strains. The numbers of the isolates correspond to those in S1 Table. With the exception noted below, the isolates analyzed here do not include those confirmed to harbor Mendelian mutations. (A) The sRNA enriched samples (35 μg) from all of the *r3b2*Δ FK506 resistant isolates lacking mutations in the target genes were obtained after incubation for 48 hours on MMC media supplemented with 1 μg/ml of FK506. sRNA blots were hybridized with an antisense-specific probe to detect *fkbA* sRNA (see Methods). *5S* rRNA probe was used as a loading control. Abundant sRNAs were detected in all of the strains with the exception of isolate 7 of the MU429 strain that was confirmed to have a Mendelian mutation. (B) Signals from blots presented in panel (A) were quantified by densitometry and the results were plotted. The bars indicate the standard deviation.

The two independent *r3b2* null mutants were grown on YPD media supplemented with FK506 and white patches of filamentous growth, indicative of the development of FK506 resistance, were observed emanating from the edge of all of the cultures. Resistant sectors developed more rapidly in the *r3b2* null mutants compared to the wild type strain (S1 Fig), which in general required longer incubation (6 to 15 days) before filamentous growth could be observed. Spores from patches with hyphal growth were transferred to YPD solid medium containing FK506 and incubated again for 3 days. This process was repeated at least one more time before analyzing the isolates to ensure a high proportion of the nuclei were carrying the mutation or the *fkbA* siRNAs in the mycelial syncytium, as described previously [4]. These resistant isolates were cross-screened on YPD medium supplemented with 100 μg/ml of rapamycin to identify those that were FK506^R^/rapa^R^ versus FK506^R^/rapa^S^ (see S1 Table), and then grown on MMC media pH 4.5 supplemented with 1 μg/ml of FK506 and genomic DNA was extracted to sequence the FK506 target genes (*fkbA* and calcineurin genes). Only 11 out of 61 FK506-resistant isolates from the R3B2 null mutant background (3/33 and 8/28 respectively from the two independent mutants) were found to have Mendelian mutations in the *fkbA* or *cnaA/cnbR* genes (Table 1 and S1 Table). The other 50 isolates were examined for the presence of *fkbA* siRNAs to analyze if FK506 resistance resulted from RNAi-dependent epimutations. Small RNAs (sRNAs) were extracted and tested for *fkbA* antisense sRNAs ([4]; see Methods). From the 50 isolates, at least 48 showed accumulation of antisense sRNA complementary to the *fkbA* mRNA indicating activation of the epimutation pathway (Fig 1A, quantified by densitometry in Fig 1B). These epimutant isolates represent almost 80% of the total number of resistant isolates (Table 1). As the wild type frequency of epimutations is usually 20 to 30% ([4]; Table 1) this result indicates an elevated epimutation frequency, similar to that caused by deletion of *rdrp1*, which encodes an RNA dependent RNA polymerase I previously demonstrated to play a role in repressing epimutant production [4]. Therefore, these findings provide evidence that, in contrast to the positive role R3B2 plays in the progression of the non-canonical Dicer-independent and canonical Dicer-dependent pathways, R3B2 negatively regulates the epimutation pathway.

**Table 1:** Frequency of epimutants/mutants in the wild type, *sexM* and RNAi mutant strains.

Strain	Background	Total analyzed	Mutations in *fkbA*	Mutations in *cnaA/cnbR*	No mutation found	Epimutants #	Epimutants %	*p*-value*
NRRL3631	Wild type	33	22	0	11	10	30.3	
R7B	*leuA-*	31	22	1	8	7	22.6	
MU412	*r3b2*Δ::*pyrG leuA-*	33	3	0	30	28	84.9	8x10^-7^
MU429	*r3b2*Δ::*pyrG leuA-*	28	7	1	20	20	71.4	0.0002
MU430	*qip*Δ::*pyrG leuA-*	23	18	3	2	0	<4.3	0.034
MU437	*rnhA*Δ::*pyrG leuA-*	30	27	1	2	0	<3.3	0.011
MU500	*rdrp3*Δ::*pyrG leuA-*	30	4	0	26	26	86.7	5x10^-7^
MU439	*rdrp3*Δ::*pyrG leuA-*	19	3	0	16	16	84.2	0.00003
MU440	*rdrp3*Δ::*pyrG leuA-*	15	0	0	15	15	100.0	3x10^-7^
MU423	*sexM*Δ::*pyrG leuA-*	15	12	0	3	3	20.0	1
MU424	*sexM*Δ::*pyrG leuA-*	16	13	0	3	3	18.8	1

*p-values were obtained based on a Fisher Exact Probability Test for a 2x2 Contingency Table, comparing each of the mutant strains individually versus the R7B strain.

### The QIP exonuclease plays a critical role in both the RNAi-dependent epimutation and the exogenously induced RNAi pathways

To better understand the interactions between the distinct RNA silencing pathways operating in *M*. *circinelloides*, we searched for other RNAi components that could exhibit a differential role in the distinct RNA degradation pathways characterized thus far.

QIP is an exonuclease that interacts with Argonaute to facilitate the activation of the RISC complex in *N*. *crassa*, and thereby promotes the production of single stranded siRNAs from duplex siRNAs [14, 15]. Interestingly, an *in silico* analysis of the *M*. *circinelloides* genome (http://genome.jgi-psf.org/Mucci2/Mucci2.home.html) revealed the existence of a putative protein with a conserved Ribonuclease H domain (ID 110517) that had 25.5% similarity and 15.2% global identity with its ortholog the QIP protein of *N*. *crassa* (both proteins were reciprocal best hits in a BlastP analysis). This putative ID 110517 ORF contains the three exonuclease 3’-5’ characteristic motifs from the DEDDh superfamily with all of the critical acidic amino acids conserved (D and E in the I motif; H and D in the II motif; H and D in the III motif, being the last histidine essential for the exonuclease activity) (S2 Fig).

We first tested if this putative QIP orthologous gene is expressed. Liquid cultures of the wild type strain were grown for 48 hours in the dark in YNB medium pH = 4.5 and total RNA was extracted from the mycelia after being exposed to light for different periods of time. The RNA was analyzed by northern blot with hybridization under stringent conditions with a *qip* specific probe. A single mRNA was detected with the expected size of the *qip* mRNA, 1.1 kb (S3 Fig), indicating that *qip* is a *bona fide* gene that is expressed during vegetative growth in *M*. *circinelloides*. Furthermore, QIP expression is independent of light in contrast to other RNAi genes (S3 Fig) [16].

**Fig 2:**
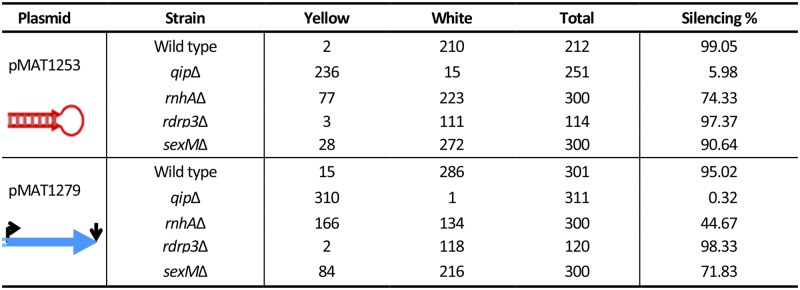
Exogenously activated silencing frequency of the *qip*, *rdrp3*, *rnhA*, and *sexM* null mutants. The color of the colonies was observed after subculture of the original transformants on YNB pH = 4.5 plates and incubation for 4 to 5 days in the light. Colonies with white patches were scored as white transformants.

A *qip*Δ null mutant (MU430) was generated by transformation, homologous recombination, and gene replacement utilizing the *pyrG* gene as the selectable marker (S4 Fig and description of plasmids and generation of deletion mutants is presented in S1 Supporting Information). PCR and Southern blot analysis confirmed that precise gene replacement and no ectopic integrations had occurred (S4 Fig and Methods). The response of the *qip*Δ mutant to different RNAi silencing triggers was examined and compared to the wild type strain to determine the role of QIP in the exogenously activated silencing response. Expression of *carB*, which encodes the enzyme phytoene dehydrogenase, is required for the production of carotenoids that endow *Mucor* with its characteristic orange pigmentation [17]; thus, in the absence of *carB* expression *Mucor* forms white colonies. The mutant and wild type strains were transformed with two different *carB* containing plasmids that are capable of triggering RNAi, plasmid pMAT1279 expresses a sense *carB* transgene [18], whereas plasmid pMAT1253 produces a *carB* RNA hairpin known to result in increased RNA silencing [19]. Both of these plasmids are self-replicating and express the *carB* alleles under the control of the strong *M*. *circinelloides gpd1* (glycerol-3-phosphate dehydrogenase) promoter. Transformation of the wild type strain with pMAT1279 or pMAT1253 resulted in a high proportion (95% and 99% with the sense and hairpin constructs, respectively) of transformed colonies that were white under illuminating conditions, indicating that *carB* expression had been silenced. However, transformation of the *qip*Δ mutant strain yielded a vast majority of orange transformants and only a few transformed colonies with mixed orange and white sectors were obtained (~0.32% and 6% with the sense and hairpin constructs, respectively), and no homogeneous white colonies were observed, indicating that the *qip*Δ mutation dramatically blocks *carB* silencing (Fig 2). These results provide evidence that, similarly to *N*. *crassa* [15], QIP has an essential role in transgene-induced silencing in *M*. *circinelloides*, further demonstrating an analogous function for both proteins. Moreover, the role of QIP in silencing is not dependent on the nature of the silencing trigger because both sense and inverted repeat transgenes showed a similar reduction in the efficiency of silencing in the *qip*Δ mutant.

**Fig 3:**
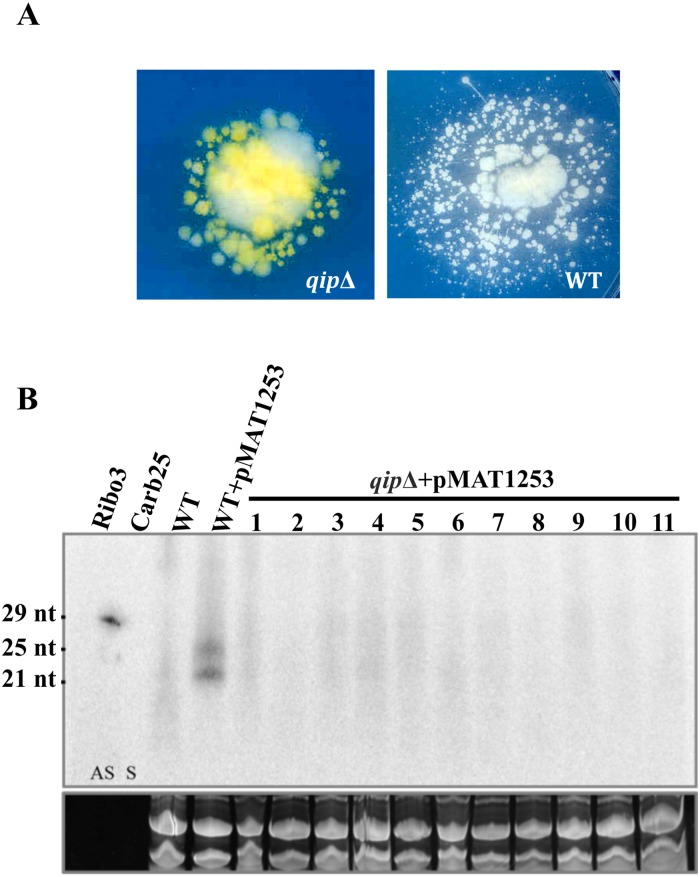
Transgene hairpin silencing is defective in *qip* null mutant. (A) Phenotype of the descendants of white colonies obtained from transformation of wild type (R7B, right) and the *qip*Δ mutant (left) with plasmid pMAT1253, which expresses a *carB* mRNA hairpin. The color of the colonies was analyzed after 48 hours growth on YNB media in the light. (B) sRNA enriched samples (50 μg) from 11 non-silenced transformants in the *qip*Δ background carrying pMAT1253 were obtained after 48 hour incubation in liquid YNB media pH = 4.5. A *carB* probe that specifically detects antisense RNA was used for hybridization in a northern blot assay. A silenced transformant with the same plasmid but in a WT background was used as a positive control. Ribo 3 (antisense, AS) and carB25 (sense, S) primers were used as size and sense markers (see Methods). tRNA were stained with ethidium bromide and served as a loading control.

Furthermore, the rare *qip*Δ *carB* transformants showing white sectors rapidly reverted to a wild type phenotype (orange) following one cycle of vegetative growth on YNB under illumination and only 13% remained white. In contrast, in accord with previous observations [18], the white *carB* silenced transformants obtained in the wild type background were more stable and 76% remained white after one cycle of vegetative growth (Fig 3A).

The ability of the *qip*Δ mutant to accumulate sRNAs was also analyzed. sRNA were extracted from the non-silenced (orange) isolates obtained following transformation of the *qip*Δ mutant with plasmid pMAT1253. Antisense sRNAs corresponding to *carB* sequences were detected by northern blot, with the silenced R7B strain serving as the positive control (see Methods). The non-silenced isolates from the *qip*Δ mutant background were unable to accumulate antisense sRNA complementary to the *carB* gene (Fig 3B), even those containing the silencing trigger and with the remainder of the RNAi pathway components intact (RdRP1, Dcl2, Ago1, RdRP2). These results indicate that QIP is required for sRNA production in the transgene-induced silencing pathway.

Next, we tested if the QIP exonuclease plays a role in the RNAi–dependent epimutation pathway. The *qip*Δ mutant strain was exposed to FK506 for several days until filamentous growth was observed emerging from the typical yeast colonies formed by *M*. *circinelloides* in the presence of the drug, as described above [4]. Of twenty-three independent FK506-resistant isolates that were analyzed, 21 harbored Mendelian mutations in the target genes (*fkbA* or calcineurin genes) (Table 1, S1 Table). The remaining two isolates were FK506-resistant but rapamycin sensitive, which indicates that these are not *fkbA* epimutants as FKBP12 is the target protein of both FK506 and rapamycin. In the absence of FKBP12, as occurs when *fkbA* is silenced, rapamycin lacks its target and has no effect on *M*. *circinelloides* [11]. This suggests that these two isolates either harbor a Mendelian mutation that has not yet been detected in the calcineurin genes or in the region of *fkbA* that affects its interaction with FK506 but not with rapamycin, or may result from a different, specific FK506-resistance mechanism. The absence of epimutants among the 23 FK506-resistant isolates and the inability of the *qip*Δ mutant to respond to RNAi silencing triggers indicate that QIP is essential for both the epimutation and the exogenously induced RNAi pathways in *M*. *circinelloides*.

### A Sad-3-like helicase is essential for the epimutation pathway but not for exogenously activated RNAi

**Fig 4:**
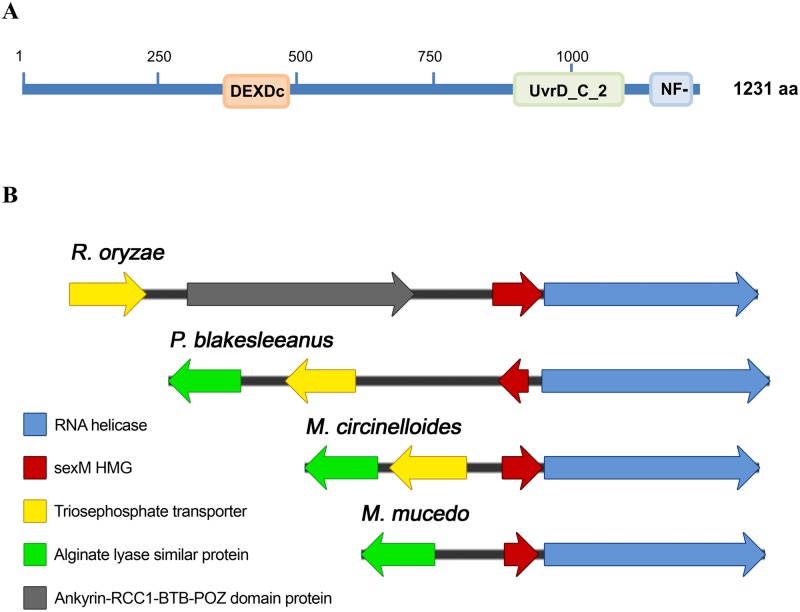
*M*. *circinelloides* RnhA protein (RNA helicase). (A) Structure of the putative *M*. *circinelloides* RnhA helicase. Three conserved domains can be found by blast with the NCBI database (http://www.ncbi.nlm.nih.gov/Structure/cdd/wrpsb.cgi): DEXDc domain, UvrD_C_2 domain, and a NF-X1-zinc-finger domain. (B) Conserved putative helicases are found in related fungal species close to the sex locus. Four different related fungal species are depicted: *Rhizopus oryzae*, *Phycomyces blakesleeanus*, *Mucor circinelloides*, and *Mucor mucedo*.

In other model fungal systems, RNA helicases are known to play important roles in RNA silencing; for example, the putative RNA helicase Sad-3 mediates meiotic silencing by unpaired DNA (MSUD) in *N*. *crassa* [20] and the *Schizosaccharomyces pombe* Hrr1 is an RNA helicase that is required for RNAi-mediated heterochromatin assembly [21]. A homology search performed with the amino acid sequences of SAD-3 and Hrr1 identified a putative RNA helicase in *M*. *circinelloides*. The coding region of this gene (named *rnhA*) is 4134 bp and its deduced protein product (Protein ID 143979) of 1231 amino acids contains the conserved UvrD-like helicase C-terminal domain with high similarity to that present in SAD-3, as well as a DEAD-like helicase superfamily domain and a putative zinc binding domain (Fig 4A). *rnhA* gene and its location are conserved throughout all of the closely related fungal species. In all of these fungi the gene is located in close proximity to the sex determining genes *sexM* and *sexP* and is the first gene in the 3’ flanking region of the sex locus. In some *Mucor* sub-species the promoter of the *rnhA* gene lies within the sex specific region of the genome (Fig 4B, [22]). *rnhA* gene expression is elevated during mating conditions, for example, during co-culture of (+) and (-) mating type cells or during vegetative growth of either mating type cell when supplemented with the sex pheromone trisporic acid (S5 Fig). However, the expression of the *rnhA* gene during vegetative growth is not as high as in mating conditions.

We found that *M*. *circinelloides* RnhA is an ortholog of NcSad-3 and SpHrr1 (S6 Fig). McRnhA and SpHrr1 are reciprocal best hits in a blast analysis. In addition, SpHrr1 and NcSad-3 are also reciprocal best hits (S6A Fig). NcSad-3 hit McRnhA first; on the other hand, McRnhA hit NcSad-3 third in the blast analysis, with two uncharacterized proteins, NCU09357 and NCU05861, as the first and second hits, respectively. Interestingly, when the three putative *N*. *crassa* RNA helicases were blasted against the *M*. *circinelloides* genome, they hit McRnhA as the most homologous protein. Subsequent phylogeny analysis indicated that the RnhA proteins in three Mucorales fungi are closely related to NcSad-3 and SpHrr1 (S6B Fig), and NCU09357 and NCU05861 are more distantly related to the RnhA/Sad-3/Hrr1 cluster. It is possible that *N*. *crassa* Sad-3, NCU09357, and NCU05861 are paralogs. The *M*. *circinelloides* genome encodes another putative RNA helicase, e.gw1.14.1.1, which is also distantly related to the RnhA/Sad-3/Hrr1 cluster. Our further analysis revealed that the three putative RNA helicases share key helicase domains (S6C Fig). These results demonstrate that *M*. *circinelloides* RnhA is an ortholog of NcSad-3 and SpHrr1. *Aspergillus fumigatus* AFUA5G09090 lies within the RhnA/Sad-3/Hrr1 cluster, suggesting a potential role of this protein in RNA silencing in this fungus.

To identify a role for the putative RnhA helicase in RNAi in *Mucor*, an *rnhA* null mutant was generated by gene replacement (S7 Fig and description of plasmids and generation of deletion mutants in S1 Supporting Information). Following 8 transformations and analysis of 357 transformants, one null mutant was obtained, which was named MU437. That correct gene replacement by homologous recombination and no ectopic integration events had occurred were confirmed by PCR and Southern blot analyses (S7 Fig and Methods).

The response of this *rnhA*Δ mutant to the activation of the different RNAi pathways was studied and compared to the wild type strain. First, the *rnhA*Δ mutant and the wild type strain were transformed with the two different silencing triggers described above, pMAT1279 and pMAT1253. Only a minor defect was observed for the exogenously activated RNA silencing pathway, as the number of white colonies in which the *carB* gene had been silenced, leading to a loss of carotene production in light, was only modestly reduced compared to the wild type: from 92 to 74% with pMAT1253 and from 79 to 45% with pMAT1279 (Fig 2). These silenced isolates did not exhibit any difference compared to the wild type silenced isolate with respect to their stability as they remained completely white after several vegetative cycles. These results suggest a minor role, if any, for this helicase in the canonical RNAi pathway operating during vegetative growth. Based on its expression pattern (S5 Fig), it is possible that RnhA may play roles in the canonical RNAi pathway during sexual development.

Next the *rnhA*Δ mutant was grown in the presence of FK506 to test for a role in epimutation-mediated development of drug resistance. After 3 weeks of growth in YPD supplemented with FK506, 30 drug resistant isolates were recovered and analyzed. None of them resulted from the activation of the epimutation pathway, as evidenced by the fact that 28 of 30 isolates contained Mendelian mutations in the FK506 target genes (*fkbA* and calcineurin genes). For only two of the isolates, were we unable to find Mendelian mutations in the FK506 target genes; one was FK506 resistant but rapamycin sensitive which, as noted above, rules out silencing of the *fkbA* gene; the second one has a probable insertion due to the lack of product from PCR amplifications of the gene region (Table 1 and S1 Table). These results indicate that despite its minor role in exogenously activated RNAi silencing, the RnhA helicase therefore plays an essential role in the epimutation mechanism of *M*. *circinelloides*. It is intriguing that the *rnhA* gene is expressed at a low level (S5 Fig) during vegetative growth, yet plays an important role in epimutation. It is therefore possible that epimutations may be evoked at a higher frequency during mating when *rnhA* is more highly expressed.

Because of the proximity of the *rnhA* gene to the sex locus (Fig 4B), we analyzed the possible contribution of the *sexM* and *sexP* genes to the RNAi silencing pathways in *M*. *circinelloides*. First we tested the effects of the *sexM*Δ null mutation (strain MU423, [23]) on silencing of the *carB* hairpin and the *carB* sense transgenes as described above. No differences were found between the wild type strain and the *sexM*Δ mutant in the exogenously activated silencing frequencies (Fig 2). Next, the *sexM*Δ null mutants MU423 and MU424 were analyzed for the development of FK506 resistance. Following incubation in the presence of FK506, 15 (from MU423) and 16 (from MU424) isolates were sequenced from which 12 and 13 isolates respectively, were found to have Mendelian mutations in the *fkbA* gene. The remaining resistant isolates showed accumulation of antisense *fkbA* sRNAs (S8 Fig). The frequency of epimutations arising in the *sexM*Δ null mutant background is therefore 19 to 20%, similar to that observed in the R7B wild type strain ([4]; Table 1). These results rule out any roles for *sexM* in silencing of exogenous transgenes or silencing mediating epimutational drug resistance during vegetative mitotic growth.

### RdRP3 represses the epimutation pathway and participates in the non-canonical RdRP-dependent Dicer-independent RNA degradation pathway

There are two known RdRP proteins in *M*. *circinelloides*. First, RdRP1 is: 1) responsible for dsRNA synthesis from sense transgenes in the canonical RNAi pathway ([18]; 2) required for the production of different classes of endogenous siRNAs ([6]; and 3) the most important element in the RdRP-dependent Dicer-independent degradation mechanism of endogenous mRNA [5]. Second, RdRP2 is essential for the transgene-induced silencing mechanism because of its prominent role in the amplification of the silencing signal [18]. However, it has only a minor role in the endogenously induced silencing and the RdRP-dependent Dicer-independent degradation mechanism [5, 6]. RdRP2 is also essential for the epimutation pathway [4], probably because amplification of the signal is necessary to activate and maintain silencing of the target gene. RdRP1 was found to have an unexpected negative role in the epimutation pathway [4]. Regardless, both mutant strains, *rdrp1*Δ and *rdrp2*Δ, were able to produce an antisense mRNA complementary to the *fkbA* mature mRNA, which is hypothesized to have a role in the activation of the epimutation pathway by binding to the *fkbA* mRNA and generating a dsRNA molecule as a trigger for RNAi [4]. Given the opposing roles of the two RdRPs in the epimutation mechanism, it seemed unlikely that they collaborate to generate the antisense mRNA and thus, the existence of a third RdRP protein was postulated.

The RdRP2 amino acid sequence was employed to blast version 2.0 of the translated genomic sequence of *M*. *circinelloides* (see above), which was not available when RdRP2 was identified [18]. A third *rdrp* gene was identified, which was named *rdrp3* (ID 80729, previously named as *r2d2*). There are five introns in the 3947 bp *rdrp3* coding region that were confirmed by cDNA sequencing and comparison with the genomic sequence. The deduced 1210 aa protein product contains the conserved RdRP domain that extends from position 407 to 944 (PFAM 4.84e-101). This putative protein shares 30% identity and 48% similarity with RdRP2 and 17% and 29%, respectively with the RdRP1 protein.

To analyze the role of this third RdRP protein in the different RNAi pathways in *M*. *circinelloides*, an *rdrp3*Δ null mutant was generated by gene replacement (S9 Fig and description of plasmids and generation of deletion mutants is presented in S1 Supporting Information). Four independent *rdrp3* deletion mutant strains were obtained (named MU438, MU439, MU440, and MU500).

One of the *rdrp3*Δ mutant strains (MU500) was transformed with the same two self-replicative silencing vectors described above, and its exogenously activated silencing response to different silencing triggers was analyzed. After subculture of the original transformants on YNB pH = 4.5 solid medium and incubation for 4 to 5 days under the light as described previously [18], the number of white/orange colonies was compared between the *rdrp3*Δ and the wild type strain. No difference was found between these two strains (Fig 2) in the activation of the transgene-induced silencing with any of the trigger constructs. These results suggest that, differently to RdRP1 and RdRP2, RdRP3 does not have a role in either the activation or the amplification leading to transgene-induced silencing.

**Fig 5:**
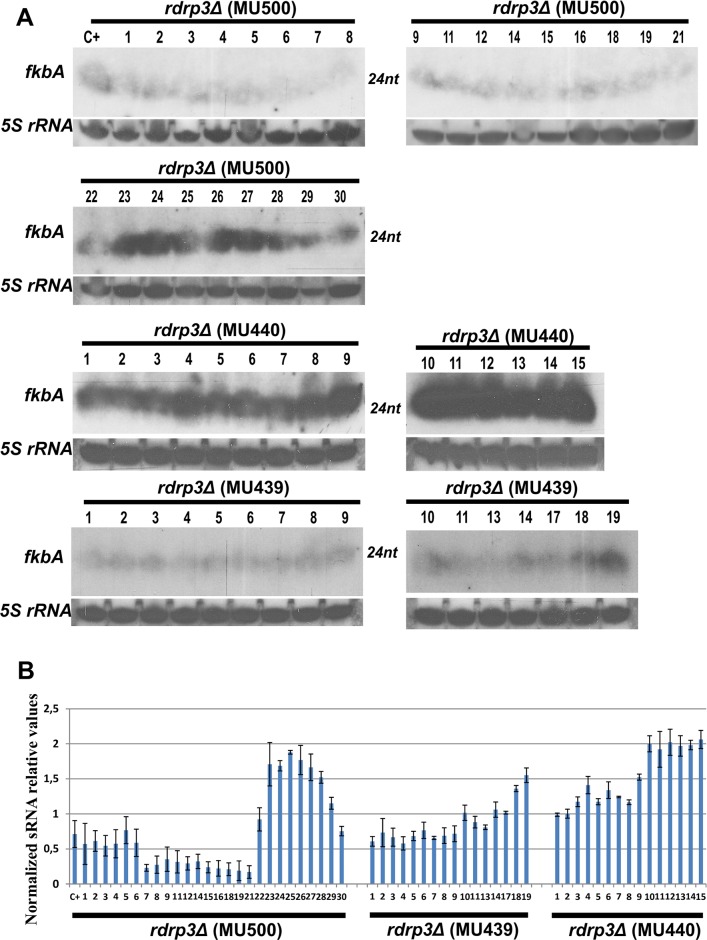
Detection of *fkbA* sRNAs in the FK506 resistant epimutant isolates of *rdrp3*Δ mutant strains. (A) The numbers of the isolates correspond to those in S1 Table. The isolates analyzed here do not include those confirmed to harbor Mendelian mutations. The sRNA enriched samples (35 μg) from the *rdrp3*Δ FK506 resistant isolates lacking mutations in the target genes were obtained after 48 hour incubation at room temperature on MMC media pH = 4.5 supplemented with 1 μg/ml of FK506. sRNA blots were hybridized with an antisense-specific probe to detect *fkbA* sRNA (see Methods). *5S* rRNA probe served as a loading control. (B) Signals from blots presented in panel (A) were quantified by densitometry and the results were plotted. The bars indicate the standard deviation.

Next, three *rdrp3*Δ mutant strains (MU439, MU440, and MU500) were exposed to FK506 to examine the role of the RdRP3 protein in the epimutation pathway. The development of FK506 resistance was more rapid in these mutants than in the wild type strain, and comparable to the result observed in the *r3b2*Δ mutant strain. Following sequencing of the target genes in the different FK506 resistant isolates, only a few (7/64) were found to harbor Mendelian mutations, while most (57 out of 64, >80%, Table 1 and S1 Table) exhibited distinctive epimutation phenotypes including production of antisense *fkbA* sRNAs (Fig 5A, quantified by densitometry in Fig 5B) to result in silencing of *fkbA* gene expression. These results illustrate that, similar to RdRP1 and R3B2, the RdRP3 protein plays a repressing role in the epimutation pathway.

**Fig 6:**
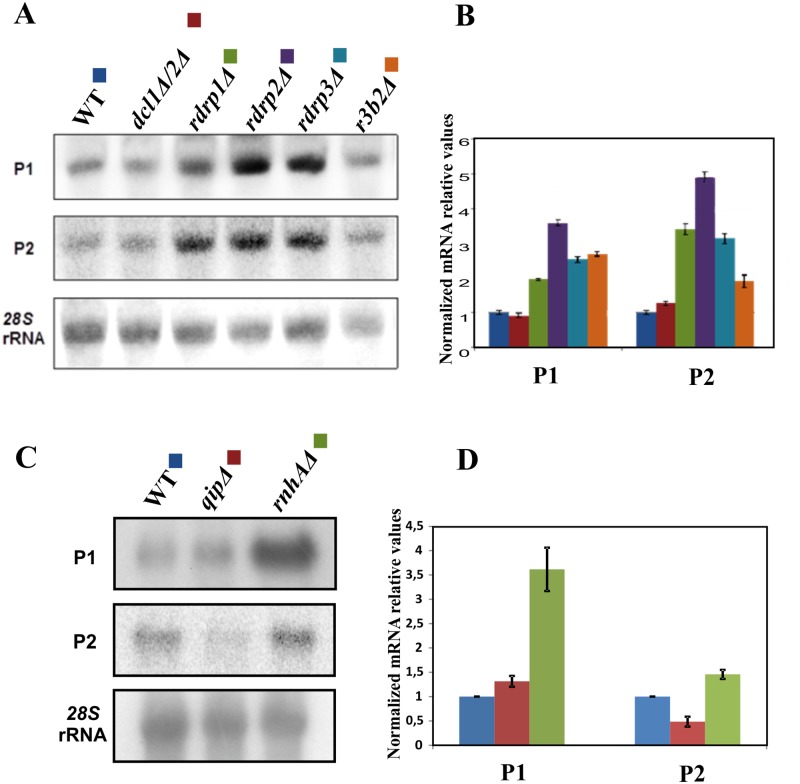
RdRP3 and RnhA participate in the *rdrp*-dependent *dicer*-independent RNA degradation pathway. (A) Accumulation of mRNAs in wild type and silencing mutants. Northern blots of high molecular weight RNAs corresponding to genes regulated by the *rdrp*-dependent RNA degradation pathway (genes P1 and P2) were carried out using total RNA (50 μg) extracted from wild type (R7B), *dcl1*Δ*/2*Δ (MU411), *r3b2*Δ (MU412), *rdrp1*Δ (MU419), *rdrp2*Δ (MU420), and *rdrp3*Δ (MU438) mutant strains grown for 24 hours in liquid MMC medium. Samples were separated in 1.2% denaturing agarose gels, transferred to membranes, and hybridized with gene specific probes (S2 Table). Target genes correspond to the following gene products: P1: ID 26072, pyruvate decarboxylase, P2: ID 92956, actin binding protein. The membranes were reprobed with a *28S* rRNA probe as loading control. Images are representative of three independent experiments. (C) Similarly, total RNA extracted from *qip* (MU430) and *rnhA* (MU437) mutant strains was analyzed in Northern blots assays. (B) and (D) Densitometric analysis of expression data shown in (A) and (C). Signal intensities were quantified and normalized to rRNA levels. All data were again normalized with respect to the expression value of the wild type strain (R7B) for each gene.

Given that RdRP1 and R3B2 are the principal components of the non-canonical RdRP-dependent Dicer-independent RNA degradation pathway, the participation of RdRP3 in this mechanism was investigated. Towards this end, we analyzed mRNA accumulation from representative loci regulated by this pathway in the wild type, *dicer1*Δ/2Δ, *r3b2*Δ, *rdrp1*Δ, *rdrp2*Δ and *rdrp3*Δ mutants by Northern blot analysis of RNA samples, isolated from cultures grown for 24 hours in liquid MMC medium. Two different target genes, P1 and P2, whose transcript accumulation is regulated by the *rdrp*-dependent *dicer*-independent degradation pathway, were selected for analysis [5]. As demonstrated before [5], lack of function of the non-canonical RNA degradation pathway in the *rdrp1*, *rdrp2*, and *r3b2* null mutants resulted in an increased mRNA accumulation of the target genes, compared to the wild type and *dicer* null mutants (Fig 6A and 6B). Similarly, both tested mRNAs were also up-regulated in the *rdrp3*Δ mutant, in which accumulation of both mRNAs increased more than two-fold compared to the wild type and *dicer* null mutant. This suggests that RdRP3 plays a crucial role in the degradation of specific mRNAs via the *rdrp*-dependent *dicer*-independent non-canonical RNA silencing pathway. In order to complete the genetic dissection of the *rdrp*-dependent *dicer*-independent non-canonical RNA silencing pathway, the role of the *qip* and *rnhA* genes that were found to be essential in the epimutant pathway was also studied in the first pathway (Fig 6C and 6D). These studies showed a significant increase in the expression of the two target genes P1 and P2 in the *rnhA* mutant, which reveals a new role for this gene in positively controlling the non-canonical pathway.

It has been shown that neither RdRP1 nor RdRP2 are individually necessary to generate the antisense strand that is thought to allow *M*. *circinelloides* to activate *fkbA* epimutation [4]. The putative role of RdRP3 in the production of antisense *fkbA* RNA was therefore tested. The *rdrp3*Δ mutant and wild type strains, as well as all of the other strains used in this study, were grown 48 hours on MMC pH = 4.5 and total RNA was extracted from the mycelia. The RNA was analyzed by northern blot, hybridizing under stringent conditions with an *fkbA* antisense specific probe (see Methods). A single mRNA with the expected size of the *fkbA* mRNA was detected in all of the strains tested (including the *rdrp3*Δ mutants) except the *fkbA*Δ mutant (S10 Fig). Because the antisense *fkbA* RNA is generated from the sense *fkbA* RNA as a template [4] it should therefore be produced by an RdRP activity. Because none of the three known RdRPs present in *Mucor* are essential for the synthesis of the antisense *fkbA* strand, the generation of this molecule is either attributable to redundant action of some combinations of RdRP1, 2, and 3 or to an as yet unknown RdRP.

### Several wild type *M*. *circinelloides f*. *circinelloides* strains exhibit an enhanced activation of the epimutation mechanism

In a previous study [4], we demonstrated that the epimutation pathway was active in not only the *M*. *circinelloides f*. *lusitanicus* lab strains (NRRL3631 and R7B) but also in a *M*. *circinelloides f*. *circinelloides* wild type strain (1006PhL), which is considered a distinct species because of mating barriers and phylogenetic separation [4]. Interestingly, this strain (1006PhL) showed an increased rate of *fkbA* epimutant silencing (90%), comparable to that obtained in the *rdrp1*Δ, *rdrp3*Δ, and *r3b2*Δ null mutants. To generalize this phenomena, we analyzed *fkbA* silencing induced by FK506 in other wild type *M*. *circinelloides f*. *circinelloides* strains: IP1873.89 [24], CNRMA03.154 [24], NRRL3615 [25], and NRRL3614 [25]. Three (IP1873.89, NRRL3615, and NRRL3614) out of the four strains showed a surprisingly high phenotypic plasticity similar to that detected in 1006PhL. The FK506 resistant isolates appeared readily and filamentous growth quickly appeared from the edge of the yeast colony in each subculture, usually from more than one place in each colony (only those isolated from different petri dishes were considered independent epimutants). Except for the CNRMA03.154 strain, which did not show any epimutant isolates, a high number of the isolates from the other three different backgrounds (84% in IP873.89, 76% in NRRL3615, and 96% in NRRL3614) showed the presence of *fkbA* sRNAs, indicating activation of the epimutation pathway (Table 2, S11 Fig, and S1 Table).

**Table 2:** Frequency of epimutants/mutants in the different *Mucor circinelloides f*. *circinelloides* strains.

Strain	Source	Total analyzed	Mutations in *fkbA*	Mutations in *cnaA/cnbR*	No mutation found	Epimutants #	Epimutants %	*p*-value*
NRRL3631	Wild type	33	22	0	11	10	30.3	
1006PhL	Human skin (Lee et al, 2014)	25	2	0	23	23	92.0	0.000002
IP1873.89	Human feces (Schwarz et al 2006)	25	4	0	21	21	84.0	0.000057
NRRL3615	Beef (Li et al 2011)	25	6	0	19	19	76.0	0.0012
CNRMA03.154	Human skin (Schwarz et al 2006)	15	13	0	2	0	<6.7	0.02
NRRL3614	Pig feces (Li et al 2011)	24	1	0	23	23	95.8	3.6x10^-7^

*p-values were obtained based on a Fisher Exact Probability Test for a 2x2 Contingency Table, comparing each of the mutant strains individually versus the NRRL3631 strain.

## Discussion

RNAi mechanisms are both complex and conserved. Despite extensive investigations into the roles of RNAi in gene regulation, novel pathways and functions continue to be discovered. In fungi, two of these novel regulatory RNAi pathways are the non-canonical RdRP-dependent Dicer-independent silencing pathway and the endogenous silencing of mRNA via epimutations, and both of these pathways operate in *M*. *circinelloides*. The first mechanism has been recently described as a non-canonical RNAi pathway involved in an mRNA degradation mechanism and its main unique feature is that the *rdrp* genes are required but not the Dicer ribonucleases. Instead, the ribonuclease activity is provided by the new protein R3B2, an RNase III-like protein that features a unique domain architecture specific to basal fungi and plants [5]. The profiling of sRNAs in the wild type and silencing mutants showed that this silencing mechanism controls the expression of target genes through the specific degradation of mRNAs by R3B2. This new role of RdRPs in the degradation of RNA could represent an evolutionary link between mRNA degradation and RNA silencing [5].

The second mechanism is an epigenetic RNAi-mediated pathway that was revealed as a new adaptive mechanism. This pathway controls phenotypic plasticity by silencing key genes to produce an epigenetically modified offspring that is better adapted to new environmental conditions. It is a remarkable mechanism that can readily respond to changes in the environment and rapidly produce a temporally adapted offspring. The main advantage of this adaptive mechanism is its reversibility [4, 7]. Thus, if changes in the environment are punctate anomalies and continued growth enables escape from a stressful condition, the new phenotype can reverse to the wild type. When the change is permanent, the temporally adapted population presents an outstanding resistance to the new condition, and thereby, a higher probability to establish new members in the population through classical mutational evolution. Genetic analysis revealed that RdRP2, Ago1, and both Dicer proteins are required for the epigenetic RNAi mechanism, whereas RdRP1 had an unexpected role constraining epimutation silencing [4].

This novel role of RdRP1 suggested that the epimutation pathway might be controlled by other genetic elements that repress the generation of epimutations under unknown conditions. To elucidate the molecular mechanisms that regulate the epimutation pathway, we conducted a genetic dissection of newly identified genes that could be involved in the different RNAi pathways of *M*. *circinelloides*. Among these genes, we have analyzed a quelling induced protein (*qip*), a Sad-3 helicase (*rnhA*), a new RdRP protein (*rdrp3*), and a novel ribonuclease involved in the non-canonical RNAi pathway (*r3b2*).

To establish the relationship between the non-canonical RNAi and the epimutation pathways, we first analyzed the ribonuclease protein R3B2. Our results showed a central role of R3B2 in all of the RNAi pathways described in *M*. *circinelloides*. Thus, in addition to the essential role of R3B2 in the regular vegetative RNAi and non-canonical RNAi pathways [5], we showed that *r3b2* mutation results in an increase in the epimutation frequency similar to that detected in *rdrp1* mutants [4]. These results indicate that both R3B2 and RdRP1 are required to activate the non-canonical RNAi pathway and suggest a constraining role of this pathway over the epimutation pathway. In support of this hypothesis, similar to *r3b2* and *rdrp1* mutants, mutant strains lacking *rdrp3*, a newly identified RDRP encoding gene, showed a blocked non-canonical pathway and an overactive epimutation pathway, indicating the same constraining role observed above. There are therefore three RdRP proteins operating in *M*. *circinelloides*. RdRP2 amplifies the different silencing signals [4, 18]. RdRP1 activates the exogenously induced silencing mechanism by generating the strand complementary to transgenes introduced in *Mucor*, and in addition represses the epimutation pathway [4, 18]. RdRP3 plays only a minor role in the canonical silencing pathway; however, it also represses the epimutation pathway.

**Fig 7:**
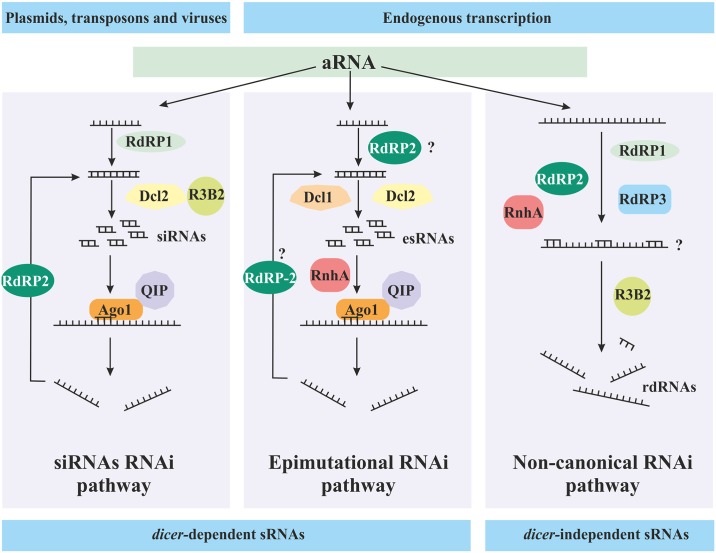
Models for the different RNAi pathways operating in *M*. *circinelloides*. The siRNA RNAi pathway (left) is a defense mechanism against invasive nucleic acids such as plasmids, transposons, and viruses [36]. Aberrant transcripts from these invasive agents are used by RdRP1 to generate dsRNA molecules, which are cleaved by Dcl2 to produce siRNAs that are transferred to Ago1. The RNase III-like protein R3B2 participates in the biogenesis of these siRNAs, although its function in this pathway is still unknown. RdRP2 generates secondary dsRNA and amplifies this pathway [18]. The epimutation RNAi pathway (middle) silences target endogenous transcripts under stress conditions to generate epimutant strains that are better adapted. In this pathway, the generation of both primary and secondary dsRNA might be under the control of RdRP2, because *rdrp2* mutants are incapable of producing epimutant strains resistant to the antifungal drug FK506 [4]. The function of RnhA might be mediated via its catalytic activity in unwinding ds-esRNAs; however, its precise role remains to be established. The non-canonical RNAi pathway (right) targets highly expressed mRNAs for degradation. RdRP1, RdRP2 [5] and RdRP3 (this study) could interact with these highly expressed mRNAs to synthesize complementary strands that signal these mRNAs for degradation by R3B2, which produces the resulting rdRNAs. RnhA (this study) has also a positive role in this pathway, although its specific function is not yet known.

Surprisingly, despite their different contributions to these silencing pathways, the three *rdrp* genes have an essential role in the non-canonical *dicer*-independent RNA silencing mechanism, as all of the *rdrp*-defective mutants show a marked reduction of mRNA degradation from the two highly expressed genes analyzed (P1 and P2, Figs 6 and 7).

The functional analysis of *rdrp1*, *rdrp3*, and *r3b2* indicates an essential role of these genes in the non-canonical RNAi pathway. Interestingly, this analysis revealed that *rdrp1*, *rdrp3*, and *r3b2* mutants exhibiting a blocked non-canonical RNAi pathway also showed an enhanced epimutation RNAi pathway. The opposing actions of the non-canonical RNAi and the epimutation pathways could be explained by a competition between these two pathways for aberrant RNA (aRNA). In this competition, success might be determined by the nature and severity of the triggering signal/stress and the ability of the activated RNAi pathway to cope with it. Similarly, a competition for aRNA between mRNA decay pathways and RNAi has been proposed in other organisms such as plants, yeasts, and flies [26–29]. In these systems, mutations in key elements of the mRNA decay machinery leads to an increased activity of the RNAi pathways, suggesting that regular clearing of aRNAs limits their entry into the silencing pathways. An alternative RNA degrading pathway was found in *Caernorhabditis elegans*, which also regulates the efficiency of the RNAi pathway, although the targets of this pathway are the mature siRNAs instead of the initial aRNA [30, 31]. In *M*. *circinelloides*, the non-canonical pathway acts as a clearing machinery degrading aRNAs and reducing the expression of the target genes whereas the epimutation pathway processes the aRNAs into signaling siRNAs that completely block the expression of the target genes.

As previously shown, the epimutation pathway provides phenotypic plasticity to allow rapid adaptation to challenging environments, which suggests that this pathway might control aRNAs under stress conditions. In this regard, we found an increased production of drug-resistant epimutants in three virulent isolates (including a human isolate) (IP1873.89, NRRL3615, and NRRL3614), which exhibit virulence in a murine model [25] (Table 2). *M*. *circinelloides f*. *circinelloides* is the *Mucor* species more frequently found associated with human infection [4]. The enhanced ability to develop FK506 resistance exhibited by three out of the four analyzed strains is consistent with our model that the epimutation pathway may enable adaptation to environmental niche conditions. More studies are needed to determine why these strains have an elevated frequency (76–96%) of epimutation when compared with *M*. *circinelloides f*. *lusitanicus*, which usually shows a frequency of 20–30% in the wild type strains.

To complete the genetic analysis of RNAi genes that could be involved in the generation of epimutants, two other genes, *qip* and *rnhA*, were functionally tested in the canonical RNAi, the epimutation pathway and the non-canonical RNAi pathway. The QIP exonuclease is an essential component of both the vegetative RNAi and MSUD pathways in *N*. *crassa* [14, 15]. Its fundamental role in the activation of the RISC complex with the production of single stranded siRNAs suggested that it also plays an important role in the different RNAi pathways of *M*. *circinelloides*. Our results demonstrate that the *M*. *circinelloides QIP* ortholog plays an essential role in both the vegetative RNAi and the epimutation pathways. A *qip* mutant is incapable of producing either regular siRNAs in response to dsRNA-trigger or drug-resistant epimutants following exposure to FK506. These results indicate that QIP could also play a role in the production of mature siRNA and activation of the RISC complex in collaboration with Ago proteins, which were shown to be essential in both the vegetative RNAi and the epimutation pathways in *M*. *circinelloides* [4, 16].

Our genetic analysis demonstrated that, similarly to QIP, the RNA helicase RnhA plays an essential role in the epimutation mechanism of *M*. *circinelloides*, as no FK506 resistant epimutants were detected in the *rnhA*Δ background. In contrast, RnhA plays a minor role in exogenously activated RNAi silencing, suggesting the existence of other RNA helicases specific to this pathway. Surprisingly, the RnhA helicase showed an important role in positively controlling the *rdrp*-dependent *dicer*-independent RNAi pathway, which adds a new component to the already complex non-canonical pathway. RNA helicases are ATP-dependent enzymes that unwind dsRNA, suggesting RnhA involvement in the separation of the two strands of the siRNAs and therefore a cooperative role with QIP and AGO proteins during the production of mature siRNAs. However, recent studies showed that the RNA helicase activity of these enzymes might not be required, and have suggested a more extensive function at every level of the RNAi pathway [32]. Our findings implicate RnhA in the production of epimutants in *M*. *circinelloides* and the *rdrp*-dependent *dicer*-independent RNAi pathway, expanding the role of RNA helicases in the RNAi mechanism. Interestingly, the *rnhA* gene is more highly expressed during mating, indicating that RnhA might have a sexual development specific role in the canonical RNA silencing that is not observed during vegetative growth. In addition, overexpression of RnhA during mating might result in a higher frequency of epimutations. Future investigations will address the roles of RnhA in the RNAi pathway during mating.

In summary, our genetic and functional analysis identified four new RNAi pathway components including R3B2, RdRP3, Qip, and RnhA, which expands the complexity of RNAi mechanisms and pathways operating in *M*. *circinelloides* (Fig 7). Remarkably, our study revealed opposing roles for the non-canonical and epimutation RNAi pathways in *M*. *circinelloides* and delineates their functions during optimal growth conditions or in generating phenotypic plasticity under stress conditions, respectively. Accordingly, we found that clinical or human associated isolates show a favored epimutation RNAi pathway, suggesting that selective pressure of the host-pathogen interaction might promote the phenotypic plasticity provided by this pathway. These results provide a comprehensive study of the mechanisms developed by emerging pathogens to acquire antifungal drug resistance, which may also impact the host-pathogen interaction.

## Materials and methods

### Strains, growth and transformation conditions

The leucine auxotroph R7B, derived from the (-) mating type *M*. *circinelloides f*. *lusitanicus* CBS 277.49 (syn. *Mucor racemosus* ATCC 1216b), was used as the WT strain. Strain MU402 that was used to generate the deletion mutants is a uracil and leucine auxotroph derived from strain R7B [33]. All of the mutants used and generated in this study were derived from strain MU402. Strains MU412 and MU429 are independent deletion mutants for the *r3b2* gene [5]. Strains MU423 and MU424 are independent deletion mutants for the *sexM* gene [25]. Strains MU419 and MU420 correspond to *rdrp1*Δ and *rdrp2*Δ mutants, respectively [18]. Cultures were grown at ~25°C on yeast extract peptone dextrose agar (YPD, 10 g/l yeast extract, 20 g/l peptone, 20 g/l dextrose, 2% agar), MMC medium pH = 4.5 (1% casamino acids, 0.05% yeast nitrogen base without amino acids and ammonium sulfate, 2% glucose) or minimal YNB media as described previously [4, 33]. Media was supplemented with uridine (200 μg/ml), leucine (20 μg/ml), FK506 (1 μg/ml) or rapamycin (100 ng/ml) when required. The cultures were routinely incubated for 48 hours except when noted otherwise or for the isolation of FK506 resistant patches, when the cultures were incubated for as long as 3 weeks in some cases. Transformation was carried out as described previously [34]. Because primary transformants are heterokaryons due to the presence of several nuclei in the protoplasts, to increase the proportion of transformed nuclei transformants were grown in selective medium for several vegetative cycles.

*M*. *circinelloides f*. *circinelloides* strains: IP1873.89 was collected from human feces and CNRMA03.154 from human skin in France. Contributed by Francois Dromer at Institut Pasteur [24]. NRRL3615 was obtained from beef in Germany and NRRL3614 from pig feces in the Netherlands. Provided by Wiley Schell from Duke University [25]. 1006PhL was collected from human skin in the USA and provided by Julie Segre at NIH [22]. Competent cells of *E*. *coli* DH5α strain were used for cloning experiments.

### FK506-resistant strain isolation

FK506 resistant isolates were obtained as described previously [4]. The different strains were grown on YPD containing 1 μg/ml of FK506 for 3 days to 3 weeks at room temperature (~25°C), until patches with hyphal growth were observed. Each isolate was derived from an independent subculture grown on a different Petri dish. The FK506 resistant isolates were grown in the presence of FK506 for at least 2 vegetative cycles to ensure that a high proportion of the nuclei in the mycelial syncytium were mutant or silenced before being analyzed.

### Generation of deletion mutants

A PCR-based strategy was used for gene cloning and generation of deletion alleles to disrupt the *qip*, *rnhA*, and *rdrp3* genes. A precise description of the constructs and the procedures used can be found in the S1 Supporting Information.

### DNA/RNA extraction and analysis

Genomic DNA from *M*. *circinelloides* mycelia grown for 48 hours on MMC media pH = 4.5 (supplemented with 1 μg/ml of FK506 when needed) was extracted with phenol-chloroform or CTAB-chloroform as previously described [4, 33]. The FK506-resistant isolates obtained from the different mutant backgrounds were verified by junction PCR for the deletion of the corresponding gene. To identify correctly integrated deletion alleles in the candidate transformants, a rapid protocol for PCR assay was used [33]. Total RNA was isolated from liquid nitrogen frozen mycelia (100 mg) using Trizol reagent following the supplier’s recommendation (Invitrogen), or the RNeasy Plant Mini Kit (QIAGEN). Small RNA was isolated as described previously [4], or using the miRVana kit (Ambion) following the supplier’s instructions. Southern and Northern blot hybridizations were carried out under stringent conditions [33]. DNA probes were labeled with [α-32P] dCTP using Ready-To-Go Labeling Beads (GE Healthcare Life Science).

For Northern blot hybridization of the *qip* gene, a 1.05 kb fragment isolated from plasmid pMAT1501 (S1 Supporting Information) by ScaI/HindIII double digestion was used. Probe ‘a’ from the *qip* gene used for Southern blot hybridization corresponds to a 1.1 kb fragment PCR amplified with primers Qip1 and Qip3 from plasmid pMAT1502 that contains the deletion allele for the *qip* gene (S1 Supporting Information). Probes 1 and 2 for the *rnhA* gene used for Southern blot hybridization correspond to 1 kb fragments that were PCR amplified using the primer pairs rnh-767/sexM1750 and rnh5672/rnh-6713, respectively. P1 and P2 probes for Northern blot hybridizations shown in Fig 5 were directly amplified from genomic DNA using specific primers (S2 Table).

Sequencing of the *fkbA* and calcineurin genes was carried out as described before [4]. *rdrp3* cDNA was generated from RNA extracted with the RNeasy Plant Mini Kit following the protocol for filamentous fungi supplied by the distributor. The FirstChoice RLM-RACE kit (Ambion) was used for cDNA amplification of the 5’ or 3’ half region of the gene. Gene-specific intron-adjacent primers used for confirmation are listed in S2 Table. Sequences were aligned with Serial-Cloner software.

Signal intensities were estimated from autoradiograms using a Shimadzu CS-9000 densitometer and the ImageJ application, an open source image analysis program (rsbweb.nih.gov/ij/).

### Small RNA analysis

Detection of sRNAs in Northern blot experiments was carried out as described [5, 35]. sRNAs (25–35 μg) were separated by electrophoresis on 15% TBE-Urea gels (Invitrogen), electrotransferred to Hybond N1 filters at 400 mA for 1 h in 0.5X TBE, and cross-linked by ultraviolet irradiation (2 pulses at 1.2x10^5^ μJ per cm^2^). Ultrasensitive hybridization buffer UltraHyb (Ambion) was used for prehybridization and hybridization. Antisense-specific riboprobes were prepared by in vitro transcription (MAXIscript transcription kit; Ambion) and labeled with [α-32P] dUTP following supplier-recommended protocols. Riboprobes were treated as described previously [7] to result in an average size of 50 nucleotides (nt). An antisense-specific probe for the *fkbA* gene was amplified from genomic DNA with primers JOHE23654 and JOHE23559 and in vitro transcribed from the T7 promotor contained within the JOHE23654 primer sequence (S2 Table). This in vitro transcribed probe was also used to detect antisense *fkbA* mRNA in the different strains by Northern blot (S10 Fig). The *carB* antisense-specific riboprobe was obtained from the in vitro transcription of linearized plasmid pMAT652 [24]. JOHE37682 and JOHE37683 were used to amplify the 5S rRNA from genomic DNA and in vitro transcribed from the T7 promotor contained in both primer sequences (S2 Table).

### Sequence analysis

Computational sequence analysis was carried out using European Bioinformatics Institute Server software (EMBL Outstation, Hinxton, U.K.), and the National Center for Biotechnology Information Server (NCBI, Bethesda, MD, USA).

## Supporting information

S1 Supporting Information Supplementary materials and methods regarding plasmid construction and generation of deletion mutants. (DOC)

S1 Fig *r3b2* null mutant develops resistance to FK506 more rapidly than the wild type strain. **A)** *r3b2*Δ (MU429) and wild type (WT, R7B) strains were incubated at room temperature on YPD media supplemented with 1 μg/ml of FK506 for up to 14 days. Each spot contains 2 x 10^4^ spores of the indicated strain. Both strains grew as a yeast colony until FK506^r^ sectors started to grow as hyphae. B) The area of the resistant sectors was measured using ImageJ software.
(DOCX)

S2 Fig Amino acid sequence of the *M*. *circinelloides* qip protein. Motifs I, II and III of the Ribonuclease H domain are in red, blue, and pink respectively. Critical residues in each motif are shown in bold and underlined.
(DOCX)

S3 Fig *M*. *circinelloides qip* gene expression. The WT strain was grown for 48 hours in the dark and incubated under the light for different lengths of time before extracting total RNA (50 μg). The RNAs were separated by electrophoresis in agarose gels and hybridized with a *qip* probe. *28S* rRNA served as a loading control.
(DOCX)

S4 Fig *M*. *circinelloides qip* disruption. **(**A) Schematic representation of the genomic region of *qip* gene in the wild type strain (R7B) and in the deletion strain obtained by homologous recombination. EcoRV restriction sites are shown in green (E) and primers used to confirm the gene replacement are shown in red. (B) The PCR product of the deletion strain generated the expected 1.9 kb fragment. (C) Southern blot of the wild type strain, *qip*Δ mutant and the pMAT1502 plasmid, all of them cleaved with the EcoRV enzyme. The Southern blot membrane was hybridized with the probe a, depicted in A. This probe corresponds to a 1.1 kb fragment PCR amplified with primers QIP1 and QIP3 from plasmid pMAT1502. C: positive control for the PCR reaction using primers included in the disruption fragment. M: GeneRuler DNA Ladder Mix (Fermentas).
(DOCX)

S5 Fig Expression of the *rnhA* gene during vegetative growth and mating. To detect the expression of the genes related to the *sex* locus, RT-PCR reactions were performed with gene specific primers. The *sexM* gene is only expressed in mating conditions, including a sexual cross between two opposite mating types [(+) x (-)], (-) or (+) mating type solo-cultured with supplementation of the sex pheromone trisporic acid. The *sexP* gene is, on the other hand, expressed during vegetative growth and mating. Expression patterns of the *sexP* and *sexM* genes in *M*. *circinelloides* are in accordance with those in other known Mucorales, including *Phycomyces blakesleeanus* and *Mucor mucedo* [37, 38] The *rnhA* gene is expressed at the highest level during mating conditions. Interestingly, although mating did not occur between *sexM*Δ x (+), the expression of the *rnhA* gene was still elevated, suggesting more genes may be involved in mating and trisporic acid synthesis in addition to the *sexM* gene.
(DOCX)

S6 Fig McRnhA is an ortholog of NcSad-3 and SpHrr1. **(**A) Blast analysis revealed that McRnhA and SpHrr1 are reciprocal best hits. In addition, SpHrr1 and NcSad-3 are also reciprocal best hits. However, interestingly, McRnhA only hit the NcSad-3 third with NCU09357 and NCU05861 as the first and second hits, although NcSad-3 hit McRnhA first. (B) A phylogenetic tree revealed that McRnhA, NcSad-3, and SpHrr1 form a cluster that is more distantly related to the two *N*. *crassa* proteins NCU09357 and NCU05861. The tree also suggests that many fungi, including *Cryptococcus neoformans* (Cn), *Ustilago maydis* (Um), *Saccharomyces cerevisiae* (Sc), and *Candida albicans* (Ca) lack an orthologous protein to the RnhA/Sad-3/Hrr1 helicases. Am: *Allomyces macrogynus*, Mc: *M*. *circinelloides*, Rd: *Rhizopus delemar*, Pb: *Phycomyces blakesleeanus*, Af: *Aspergillus fumigatus*. Bootstrap is 1000 and the scale is one substitution per position. (C) Sad-3 has 1160 amino acids, Hrr1 has 999 amino acids, and RnhA has 1231 amino acids. The three proteins share a common domain as RNA helicases. In particular, RnhA is predicted to have an additional NF-X1-zinc finger domain.
(DOCX)

S7 Fig *M*. *circinelloides rnhA* disruption. **(**A) Schematic representation of the genomic region of the *rnhA* gene in the WT strain (MU402) and in the deletion strain obtained by homologous recombination. Primers used for construction of the disruption fragment and to confirm gene replacement are shown in red. (B) The PCR product of the deletion strain generated the expected 1 and 1.2 kb from the 3’ and 5’ junctions respectively (a and b). Two control PCRs amplified an internal 1 kb fragment of the disruption fragment (c) and a control 1 kb fragment from a different genomic region amplified using primers ago23 and ago26 (d). M: GeneRuler DNA Ladder Mix (Fermentas). (C) Representation of the wild type (WT) and *rnhA*Δ mutant (MUT) restriction enzyme map used to confirm the correct insertion of the disruption fragment by Southern blot shown in D. (D) Southern blot of the wild type strain (R7B) and the *rnhA*Δ mutant cleaved with ClaI, EcoRV, and NcoI enzymes. The Southern blot membrane was hybridized with probes P1 and P2, depicted in A and C. These probes correspond to 1 kb fragments PCR amplified with primers rnh-767/rnh1759 and rnh5672/rnh-6713, respectively. M: GeneRuler DNA Ladder Mix (Fermentas).
(DOCX)

S8 Fig Confirmation of the presence of *fkbA* sRNAs in the FK506 epimutant resistant isolates of *sexM*Δ mutant strains. The numbers of the isolates correspond to those in S1 Table. sRNA enriched samples (35 μg) were obtained after 48 hours incubation on MMC media supplemented with 1 μg/ml of FK506 at room temperature from the two *sexM*Δ mutant strains MU423 and MU424. sRNA blots were hybridized with an antisense-specific probe to detect *fkbA* sRNA (see Methods). A *5S* rRNA probe served as a loading control.
(DOCX)

S9 Fig *M*. *circinelloides rdrp3* disruption. **(**A) Schematic representation of the genomic region of the *rdrp3* gene in the wild type strain (MU402) and in the deletion strains obtained by homologous recombination. Primers used for construction of the disruption fragment and to confirm gene replacement are shown in red. (B) The PCR product of the deletion strains generated the expected 1.28 kb fragment (a). Two control PCRs amplified an internal 1.15 kb fragment of the disruption fragment (b) and a control 1.5 kb fragment from a different genomic region amplified using primers ago23 and ago26 (c). M: GeneRuler DNA Ladder Mix (Fermentas).
(DOCX)

S10 Fig Detection of the antisense *fkbA* mRNA in the different mutants and strains. Northern blots were carried out using total RNA (50 μg) extracted from the indicated wild types and mutant strains grown for 48 hours on MMC medium at room temperature. Samples were separated in a 1.2% denaturing agarose gel, transferred to membranes, and hybridized with gene specific probes (S2 Table). *18S* rRNA was used as a loading control.
(DOCX)

S11 Fig *M*. *circinelloides f*. *circinelloides* wild type strains show an increased activation of the epimutation pathway. The numbers of the isolates correspond to those in S1 Table. With the two exceptions noted below, the isolates analyzed here do not include those confirmed to harbor Mendelian mutations. The sRNA enriched samples (35 μg) from all the FK506 resistant isolates lacking mutations in the target genes were obtained after 48 hour incubation on MMC media supplemented with 1 μg/ml of FK506. sRNA blots were hybridized with an antisense-specific probe to detect *fkbA* sRNA (see Methods). *5S* rRNA probe was used as a loading control. Abundant sRNAs were detected in all of the isolates with the exception of IP1873.89 #19 and NRRL3615 #18 that were confirmed to have a Mendelian mutation afterwards.
(DOCX)

S1 Table Identification of FK506 and rapamycin resistant strains. (XLSX)

S2 Table Oligonucleotides used for cloning and functional analysis. ^1^Nucleotides in red indicate restriction sites used for cloning or to release the disruption fragment from the deletion vectors. Nucleotides in bold indicate the sequence of the T7 promotor used for in vitro transcription of RNA probes.
(DOCX)
