# Supplementary material for: A non-canonical RNA degradation pathway suppresses RNAi-dependent epimutations in the human fungal pathogen Mucor circinelloides
Source: PLoS Genet. 2017 Mar 24;13(3):e1006686. doi: 10.1371/journal.pgen.1006686 (PMC5384783; doi:10.1371/journal.pgen.1006686)
Supplement: S1 Supporting Information — (DOC) [file pgen.1006686.s001.doc]

**S1 Supporting Information**

**Supplementary Materials and Methods**

**Plasmids**

Plasmid pMAT1501 contains a 3.34 kb genomic fragment that includes the *qip* coding region and adjacent sequences (1042 bp upstream and 1200 bp downstream). It was amplified from genomic DNA using oligonucleotides Qip1 and Qip2 (S2 Table), which contains restriction sites for XbaI and SalI, respectively, and cloned into pBluescript II SK+. Plasmid pMAT1702 harbors a 5.94 kb DNA fragment containing the complete *rnhA* gene and adjacent sequences cloned into pGEM-T vector. The fragment was amplified using primers rnh-767 and rnh-6713 (S2 Table), which include SphI restriction sites for cloning, and PstI and SacI sites, respectively, for isolation of the disruption fragment (see below). Plasmid pMAT1341 contains a 5.1 kb genomic fragment that includes the entire *rdrp3* locus and flanking sequences. It was generated by PCR-amplification using primers rdrp3clon1, which includes a SacI restriction site, and rdrp3clon2 (S2 Table). This fragment was cloned into the pBluescript II SK+ vector using the restriction site SacI and an internal KpnI site located at its 3’ end.

Plasmid pMAT1502 was constructed to disrupt the *qip* gene. It contains the *M. circinelloides* *pyrG* gene (which complements the uracil auxotrophy of the MU402 strain) flanked by 1 kb adjacent to the *qip* gene to allow homologous recombination. In brief, plasmid pMAT1501 was inverse PCR-amplified using oligonucleotides Qip3 and Qip4 (S2 Table), which contain SpeI and PstI restriction sites, respectively. These primers amplified a 5.5 kb fragment containing the vector sequence flanked by *qip* gene adjacent sequences. The double digested SpeI/PstI fragment was ligated with a 3.4 kb fragment isolated from pEMP1, which includes a wild type allele of the *M. circinelloides* *pyrG* gene, to yield plasmid pMAT1502, in which *pyrG* substituted for 1024 bp of the *qip* gene coding region. A 4.9 kb replacement fragment was released from plasmid pMAT1502 by AatII and BsptI double digestion and introduced into MU402 by transformation. Plasmid pMAT1703 was constructed to disrupt the *rnhA* gene following a similar strategy, using plasmid pMAT1702 as a template for inverse PCR amplification with primers rnh5672 and rnh1759, which contain BglII restriction sites (S2 Table). The amplified fragment was BglII digested and ligated with the 3.4 kb *pyrG* fragment to yield plasmid pMAT1702. A 5.4 kb replacement fragment harboring the *pyrG* gene flanked by 1.0 kb of *rnhA* upstream and downstream sequences was released from plasmid pMAT1702 by PstI and SacI digestion, and introduced into MU402 by transformation. Finally, pMAT1345 was generated to disrupt the *rdrp3* gene, using plasmid pMAT1341 as a template for inverse PCR amplification with primers rdrp3del1 and rdrp3del2 (S2 Table), which include BamHI restriction sites. The 5.1 kb amplified fragment was digested with BamHI and ligated with the 3.4 kb *pyrG* fragment to give plasmid pMAT1343. A 5.4 kb replacement fragment harboring the *pyrG* gene flanked by 1.0 kb of upstream and downstream *rdrp3* sequences isolated from pMAT1343 was cloned into pUC18 to yield plasmid pMAT1345, released from this plasmid by SacI/KpnI digestion and introduced into MU402 by transformation.

**Generation of deletion mutants**

Deletion vectors pMAT1502, pMAT1702, and pMAT1345 were designed to disrupt the *qip*, *rnhA,* and *rdrp3* genes, respectively. The MU402 strain (Ura^-^, Leu^-^) was transformed with restriction fragments from each plasmid containing the *pyrG* gene flanked by sufficient sequences of the genes to allow homologous recombination. Ura^+^ transformants were grown in selective medium for several vegetative cycles to increase the proportion of transformed nuclei, because primary transformants are heterokaryons due to the presence of several nuclei in the protoplasts. Homokaryotic transformants were PCR analyzed to distinguish homologous from ectopic integrations, using the primer pairs pyrGZ/Qip-5 (*qip* gene) (S5 Fig), pyrgR2/H-up and pyrGF2/H-down (5’ and 3’ ends of *rnhA* gene) (S8 Fig), and pyrgR2/Rdrp3-5-new (*rdrp3* gene) (S10 Fig) (S2 Table) to identify homologous integration events in the corresponding loci. Four independent transformants obtained with the *rdrp3* disruption fragment amplified the expected PCR fragment and they were named MU438, MU439, MU440 and MU500. In the *rdrp3* mutant allele 3.1 kb of the *rdrp3* coding region is replaced with the *pyrG* gene. Only one out of 37 and one out of 357 transformants obtained with the *qip* and *rnhA* disruption fragments, respectively, amplified the expected fragments, and the correct disruption of the corresponding genes was confirmed by Southern analysis (S5 and S8 Figs). Digested DNA from the transformants was hybridized with specific probes that recognized both wild type and mutant alleles but could discriminate between them (probe *a* for the *qip* gene, probes 1 and 2 for the *rnhA* gene, S5 and S8 Figs). The selected transformants showed the expected fragments derived from the corresponding deletion alleles and the absence of the wild type ones, confirming that those mutants have successfully replaced the wild type allele (S5 and S8 Figs). Those mutants were named MU430 (*qip*) and MU437 (*rnhA*). The mutant allele in MU430 replaced 1.02 kb of the *qip* coding region with the *pyrG* gene. The mutant allele in MU437 eliminated 3.9 kb of the *rnhA* coding region and replaced it with the *pyrG* gene. All of these mutant strains were considered null mutants.
