# Supplementary figures and images for: A non-canonical RNA degradation pathway suppresses RNAi-dependent epimutations in the human fungal pathogen Mucor circinelloides
Source: PLoS Genet. 2017 Mar 24;13(3):e1006686. doi: 10.1371/journal.pgen.1006686 (PMC5384783; doi:10.1371/journal.pgen.1006686)

Supplemental Figure 1

A


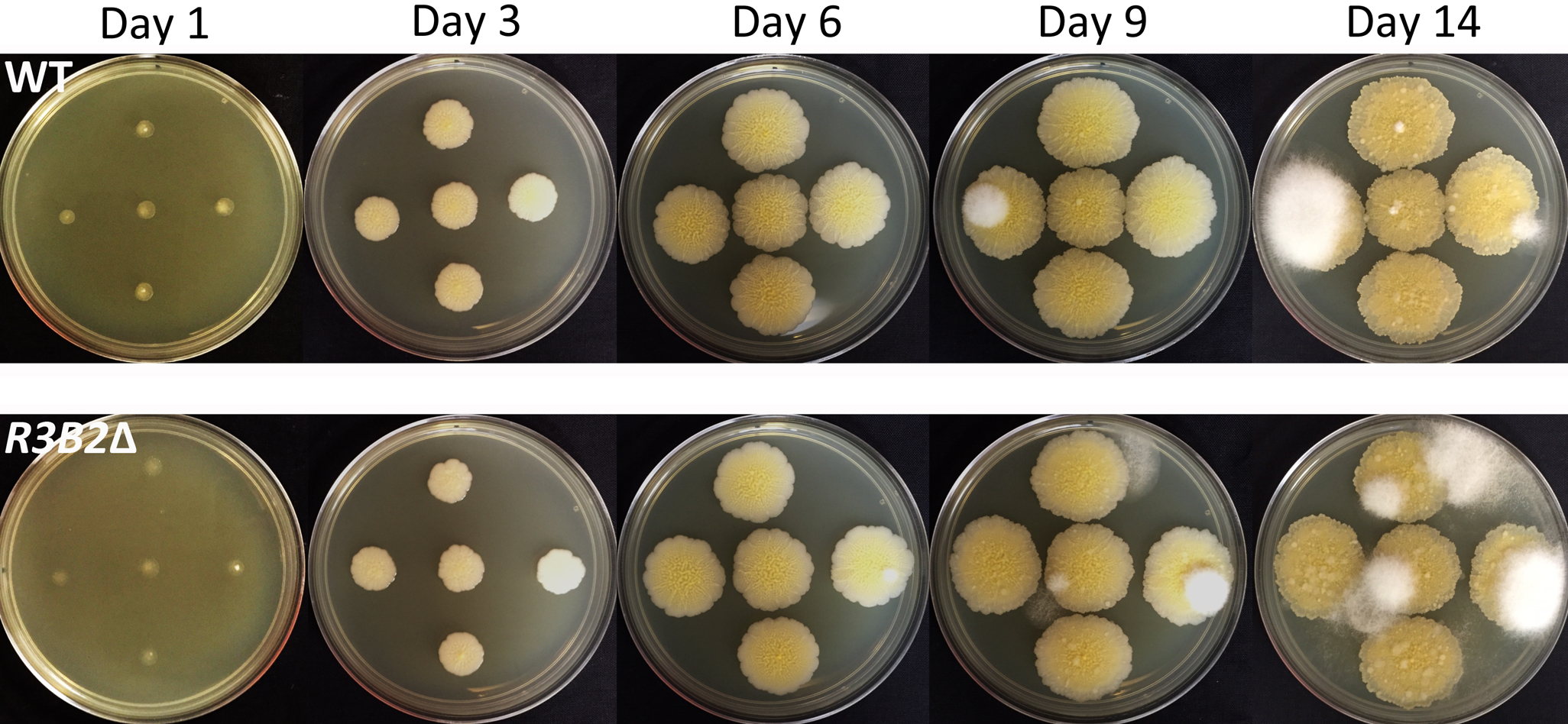


*r3b2*Δ

WT

B

**
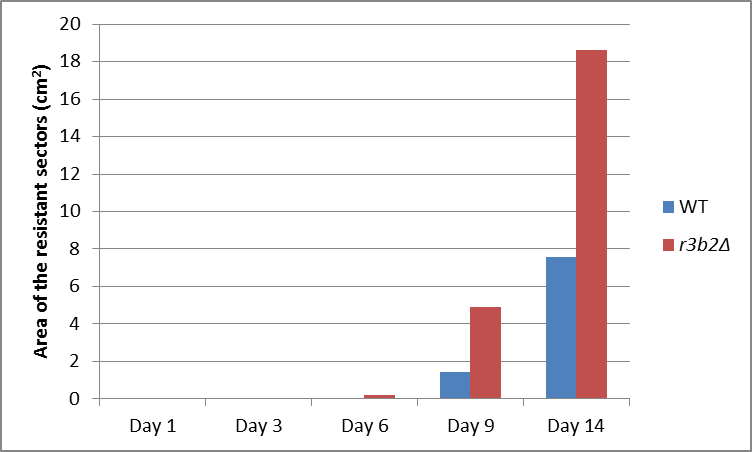
**

Supplement: S1 Fig — A) r3b2Δ (MU429) and wild type (WT, R7B) strains were incubated at room temperature on YPD media supplemented with 1 μg/ml of FK506 for up to 14 days. Each spot contains 2 x 10^4^ spores of the indicated strain. Both strains grew as a yeast colony until FK506r sectors started to grow as hyphae. B) The area of the resistant sectors was measured using ImageJ software. (DOCX) [file pgen.1006686.s002.docx]

Supplemental Figure 3

**
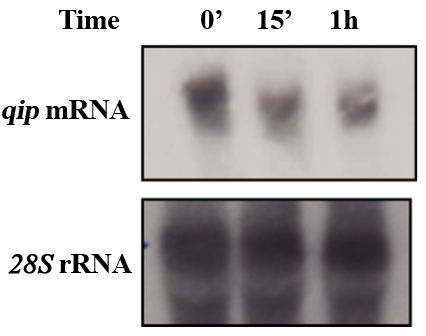
**

Supplement: S3 Fig — The WT strain was grown for 48 hours in the dark and incubated under the light for different lengths of time before extracting total RNA (50 μg). The RNAs were separated by electrophoresis in agarose gels and hybridized with a qip probe. 28S rRNA served as a loading control. (DOCX) [file pgen.1006686.s004.docx]

Supplemental Figure 4

**A**


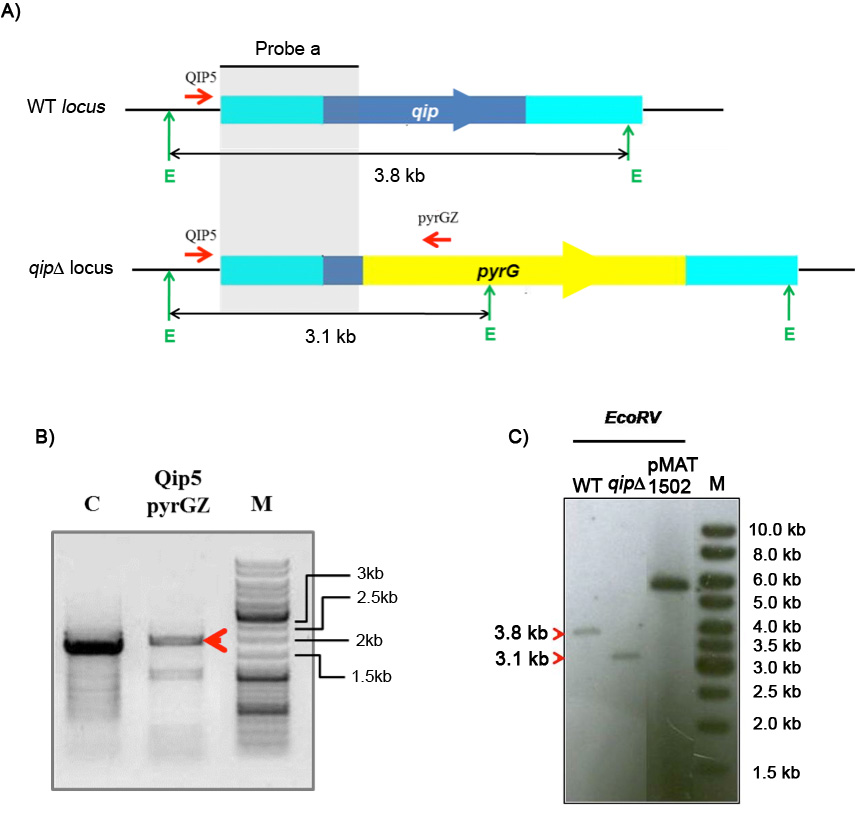


**B**

**C**

Supplement: S4 Fig — (A) Schematic representation of the genomic region of qip gene in the wild type strain (R7B) and in the deletion strain obtained by homologous recombination. EcoRV restriction sites are shown in green (E) and primers used to confirm the gene replacement are shown in red. (B) The PCR product of the deletion strain generated the expected 1.9 kb fragment. (C) Southern blot of the wild type strain, qipΔ mutant and the pMAT1502 plasmid, all of them cleaved with the EcoRV enzyme. The Southern blot membrane was hybridized with the probe a, depicted in A. This probe corresponds to a 1.1 kb fragment PCR amplified with primers QIP1 and QIP3 from plasmid pMAT1502. C: positive control for the PCR reaction using primers included in the disruption fragment. M: GeneRuler DNA Ladder Mix (Fermentas). (DOCX) [file pgen.1006686.s005.docx]

Supplemental Figure 6

**
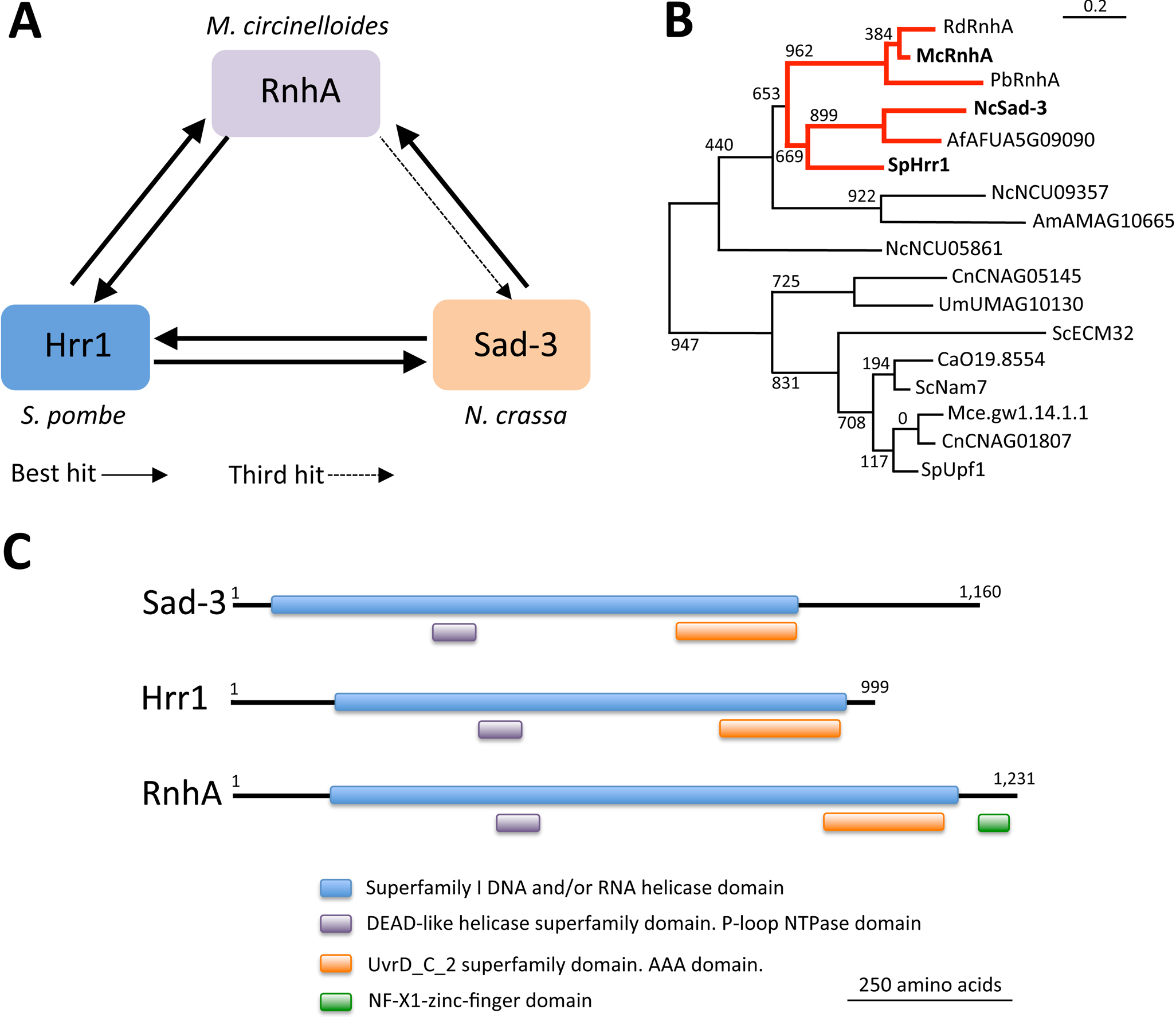
**

Supplement: S6 Fig — (A) Blast analysis revealed that McRnhA and SpHrr1 are reciprocal best hits. In addition, SpHrr1 and NcSad-3 are also reciprocal best hits. However, interestingly, McRnhA only hit the NcSad-3 third with NCU09357 and NCU05861 as the first and second hits, although NcSad-3 hit McRnhA first. (B) A phylogenetic tree revealed that McRnhA, NcSad-3, and SpHrr1 form a cluster that is more distantly related to the two N. crassa proteins NCU09357 and NCU05861. The tree also suggests that many fungi, including Cryptococcus neoformans (Cn), Ustilago maydis (Um), Saccharomyces cerevisiae (Sc), and Candida albicans (Ca) lack an orthologous protein to the RnhA/Sad-3/Hrr1 helicases. Am: Allomyces macrogynus, Mc: M. circinelloides, Rd: Rhizopus delemar, Pb: Phycomyces blakesleeanus, Af: Aspergillus fumigatus. Bootstrap is 1000 and the scale is one substitution per position. (C) Sad-3 has 1160 amino acids, Hrr1 has 999 amino acids, and RnhA has 1231 amino acids. The three proteins share a common domain as RNA helicases. In particular, RnhA is predicted to have an additional NF-X1-zinc finger domain. (DOCX) [file pgen.1006686.s007.docx]

Supplemental Figure 7

**A**


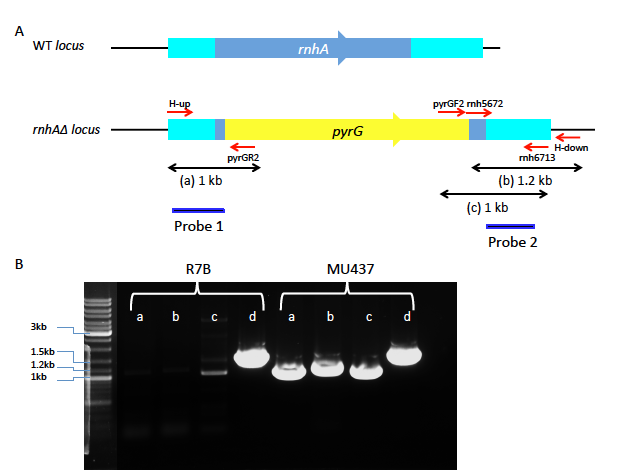


**3 kb**

**1.5 kb**

**1.2 kb**

**1 kb**

***rnhA*Δ locus**

**WT locus**

**B**

**
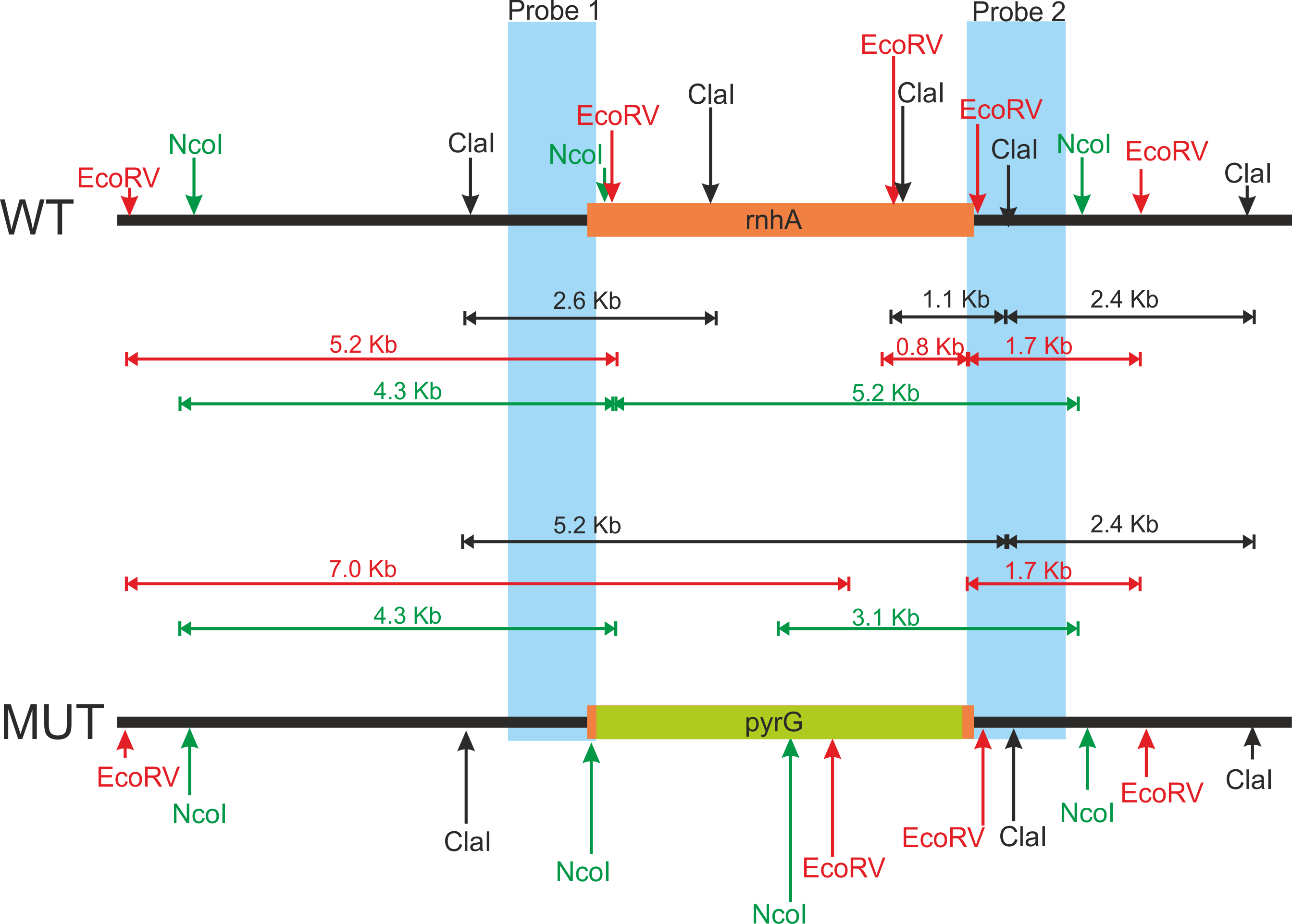
**

**C**

**D**

Supplement: S7 Fig — (A) Schematic representation of the genomic region of the rnhA gene in the WT strain (MU402) and in the deletion strain obtained by homologous recombination. Primers used for construction of the disruption fragment and to confirm gene replacement are shown in red. (B) The PCR product of the deletion strain generated the expected 1 and 1.2 kb from the 3’ and 5’ junctions respectively (a and b). Two control PCRs amplified an internal 1 kb fragment of the disruption fragment (c) and a control 1 kb fragment from a different genomic region amplified using primers ago23 and ago26 (d). M: GeneRuler DNA Ladder Mix (Fermentas). (C) Representation of the wild type (WT) and rnhAΔ mutant (MUT) restriction enzyme map used to confirm the correct insertion of the disruption fragment by Southern blot shown in D. (D) Southern blot of the wild type strain (R7B) and the rnhAΔ mutant cleaved with ClaI, EcoRV, and NcoI enzymes. The Southern blot membrane was hybridized with probes P1 and P2, depicted in A and C. These probes correspond to 1 kb fragments PCR amplified with primers rnh-767/rnh1759 and rnh5672/rnh-6713, respectively. M: GeneRuler DNA Ladder Mix (Fermentas). (DOCX) [file pgen.1006686.s008.docx]

Supplemental Figure 8


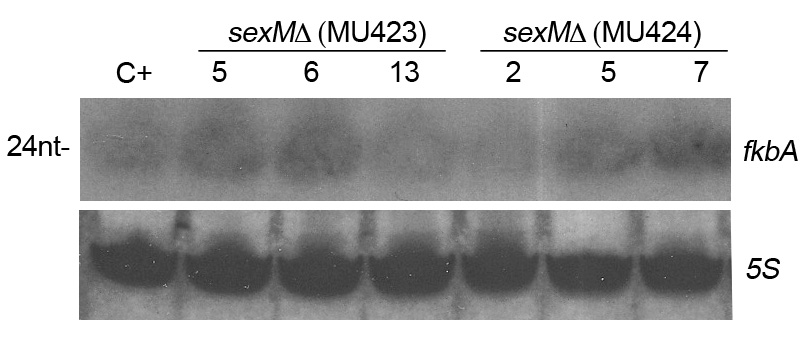

Supplement: S8 Fig — The numbers of the isolates correspond to those in S1 Table. sRNA enriched samples (35 μg) were obtained after 48 hours incubation on MMC media supplemented with 1 μg/ml of FK506 at room temperature from the two sexMΔ mutant strains MU423 and MU424. sRNA blots were hybridized with an antisense-specific probe to detect fkbA sRNA (see Methods). A 5S rRNA probe served as a loading control. (DOCX) [file pgen.1006686.s009.docx]

Supplemental Figure 10

.89


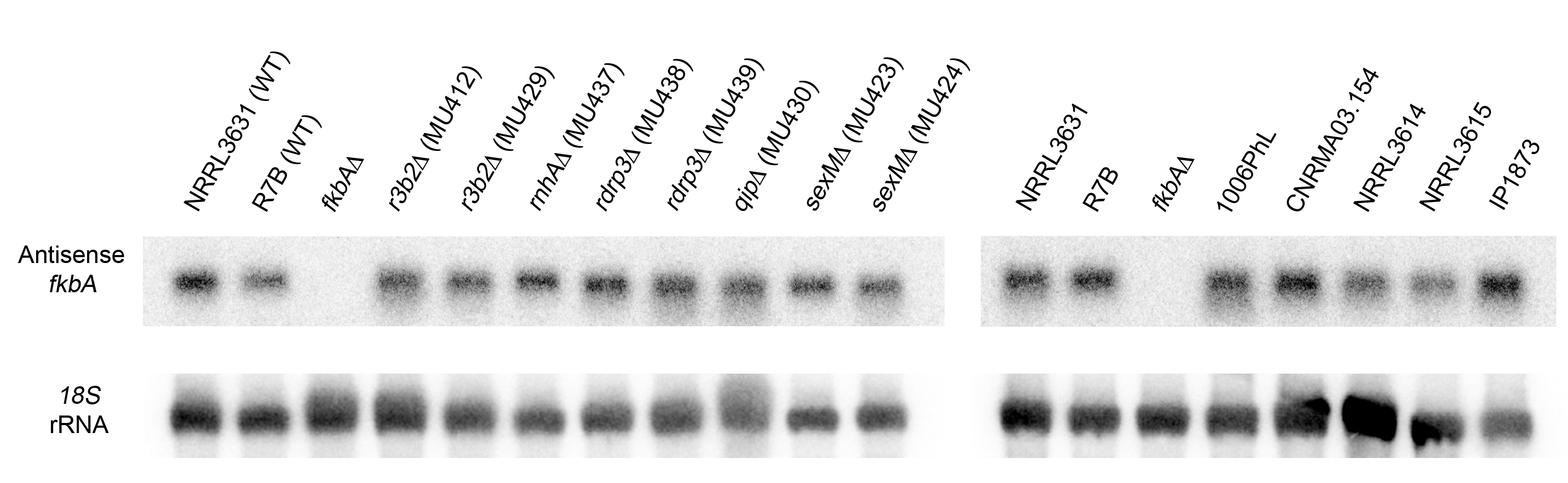

Supplement: S10 Fig — Northern blots were carried out using total RNA (50 μg) extracted from the indicated wild types and mutant strains grown for 48 hours on MMC medium at room temperature. Samples were separated in a 1.2% denaturing agarose gel, transferred to membranes, and hybridized with gene specific probes (S2 Table). 18S rRNA was used as a loading control. (DOCX) [file pgen.1006686.s011.docx]

Supplemental Figure 11

**
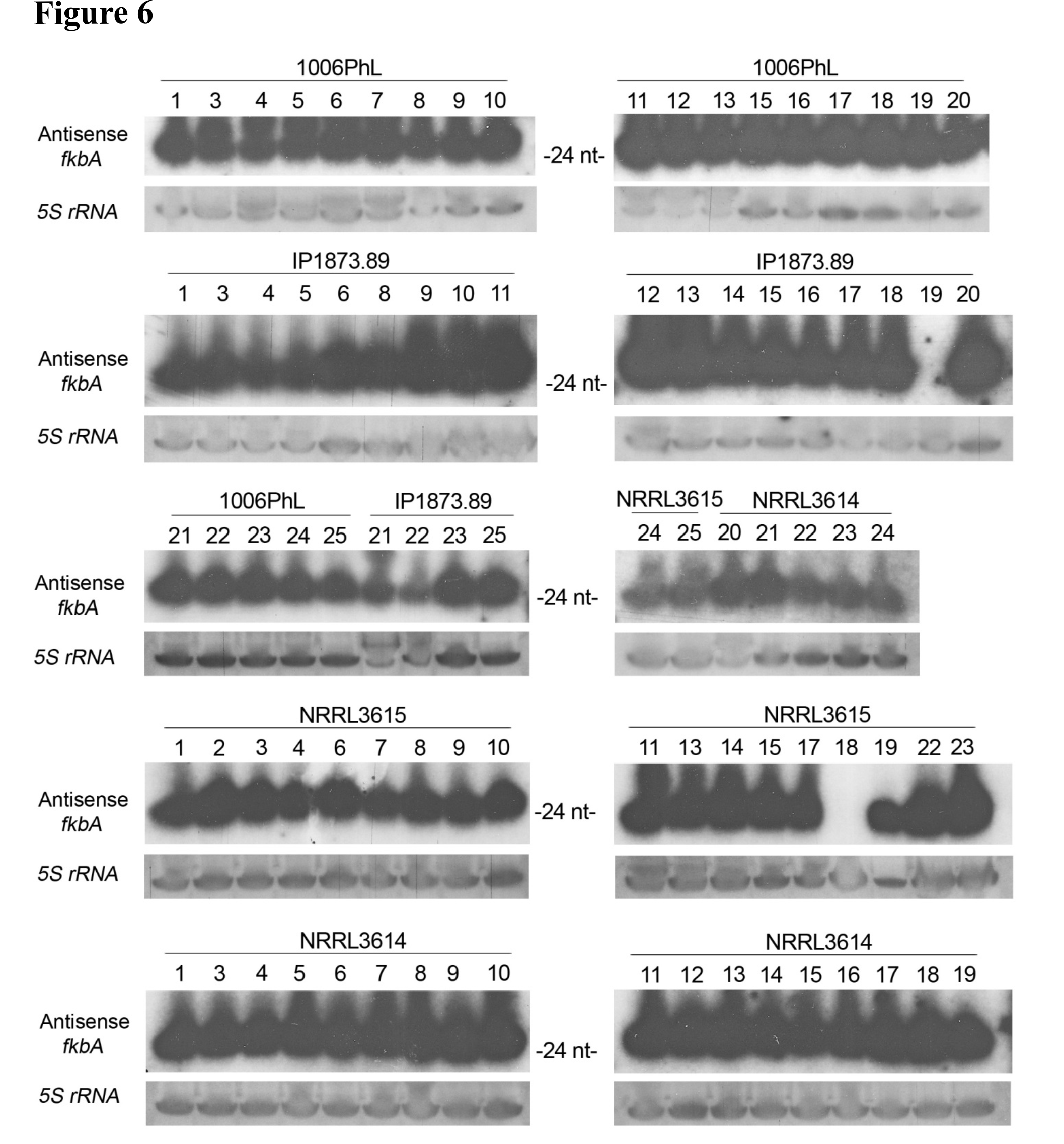
**

Supplement: S11 Fig — The numbers of the isolates correspond to those in S1 Table. With the two exceptions noted below, the isolates analyzed here do not include those confirmed to harbor Mendelian mutations. The sRNA enriched samples (35 μg) from all the FK506 resistant isolates lacking mutations in the target genes were obtained after 48 hour incubation on MMC media supplemented with 1 μg/ml of FK506. sRNA blots were hybridized with an antisense-specific probe to detect fkbA sRNA (see Methods). 5S rRNA probe was used as a loading control. Abundant sRNAs were detected in all of the isolates with the exception of IP1873.89 #19 and NRRL3615 #18 that were confirmed to have a Mendelian mutation afterwards. (DOCX) [file pgen.1006686.s012.docx]
