# Supplementary material for: A non-canonical RNA degradation pathway suppresses RNAi-dependent epimutations in the human fungal pathogen Mucor circinelloides
Source: PLoS Genet. 2017 Mar 24;13(3):e1006686. doi: 10.1371/journal.pgen.1006686 (PMC5384783; doi:10.1371/journal.pgen.1006686)
Supplement: S2 Fig — Motifs I, II and III of the Ribonuclease H domain are in red, blue, and pink respectively. Critical residues in each motif are shown in bold and underlined. (DOCX) [file pgen.1006686.s003.docx]

Supplemental Figure 2

MEKEYCLGWHLVYDALRDYYYSYGESAQKRFNHFYDPVHFFAPESLTVGIPLSIGAHAGTCEVVMPRHIYEQLVSFIHQEIPEVPEFQLETLTPVEYEIIERFEAFASNVLSKSHQKAKKKTKQLNRIRQAHEEAALAKRLVNSSNYVFVSI**D**I**E**AYEKDHSILLEIGWSMYDASTNTCMDQHYINDQYRHLLNGQFVEDQKEKFNYGTSVWCSLKQALIELRKDLDWAVKRDGGFVLVG**H**GLDS**D**LKYLAKQHFLWPGRHGGDVASVQESANVAILNTDTIYGSSINDLHNPPSLGKTLALFGIDTWNL**H**NAGN**D**AHYTLLLLLKLVHDHCDI
