# Supplementary material for: A non-canonical RNA degradation pathway suppresses RNAi-dependent epimutations in the human fungal pathogen Mucor circinelloides
Source: PLoS Genet. 2017 Mar 24;13(3):e1006686. doi: 10.1371/journal.pgen.1006686 (PMC5384783; doi:10.1371/journal.pgen.1006686)
Supplement: S5 Fig — To detect the expression of the genes related to the sex locus, RT-PCR reactions were performed with gene specific primers. The sexM gene is only expressed in mating conditions, including a sexual cross between two opposite mating types [(+) x (-)], (-) or (+) mating type solo-cultured with supplementation of the sex pheromone trisporic acid. The sexP gene is, on the other hand, expressed during vegetative growth and mating. Expression patterns of the sexP and sexM genes in M. circinelloides are in accordance with those in other known Mucorales, including Phycomyces blakesleeanus and Mucor mucedo [37, 38] The rnhA gene is expressed at the highest level during mating conditions. Interestingly, although mating did not occur between sexMΔ x (+), the expression of the rnhA gene was still elevated, suggesting more genes may be involved in mating and trisporic acid synthesis in addition to the sexM gene. (DOCX) [file pgen.1006686.s006.docx]

Supplemental Figure 5


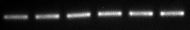


1 2 3 4 5

1. (+) only with trisporic acid (100μg)
2. (-) only with trisporic acid (100μg)
3. *sexM*$\Delta$ x (-): no mating occurs
4. *sexM*$\Delta$ x (+): no mating occurs
5. (+) mating type only


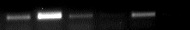

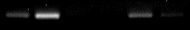

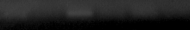


actin

*rnhA*

*sexP*

*sexM*


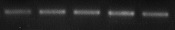

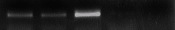

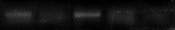

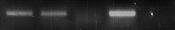


6 7 8 9 10

6. (-) mating type only

7. (+) mating type only

8. Ectopic *sexP* in *sexM*$\Delta$ x (-)

9. *sexM*$\Delta$ x (+): no mating occurs

10. Mating (+) x (-)
