# Supplementary material for: A non-canonical RNA degradation pathway suppresses RNAi-dependent epimutations in the human fungal pathogen Mucor circinelloides
Source: PLoS Genet. 2017 Mar 24;13(3):e1006686. doi: 10.1371/journal.pgen.1006686 (PMC5384783; doi:10.1371/journal.pgen.1006686)
Supplement: S9 Fig — (A) Schematic representation of the genomic region of the rdrp3 gene in the wild type strain (MU402) and in the deletion strains obtained by homologous recombination. Primers used for construction of the disruption fragment and to confirm gene replacement are shown in red. (B) The PCR product of the deletion strains generated the expected 1.28 kb fragment (a). Two control PCRs amplified an internal 1.15 kb fragment of the disruption fragment (b) and a control 1.5 kb fragment from a different genomic region amplified using primers ago23 and ago26 (c). M: GeneRuler DNA Ladder Mix (Fermentas). (DOCX) [file pgen.1006686.s010.docx]

Supplemental Figure 9
