# Supplementary material for: A non-canonical RNA degradation pathway suppresses RNAi-dependent epimutations in the human fungal pathogen Mucor circinelloides
Source: PLoS Genet. 2017 Mar 24;13(3):e1006686. doi: 10.1371/journal.pgen.1006686 (PMC5384783; doi:10.1371/journal.pgen.1006686)
Supplement: S2 Table — 1Nucleotides in red indicate restriction sites used for cloning or to release the disruption fragment from the deletion vectors. Nucleotides in bold indicate the sequence of the T7 promotor used for in vitro transcription of RNA probes. (DOCX) [file pgen.1006686.s014.docx]

**Table S2. Oligonucleotides used for cloning and functional analysis.**

| **Genes** | **Name** | **Sequence^1^** |
| --- | --- | --- |
| ***qip*** | Qip1 | 5’ GCGGGTCTAGATAGCGGCTGCTGATGCGCTTCG 3’ |
|  | Qip2 | 5’ GCGGGGTCGACATACGCCATGCAGGCGTGCGCG 3’ |
|  | Qip3 | 5’ CCGCCACTAGTCCGTTTCTGTGCCGACTCACCG 3’ |
|  | Qip4 | 5’ CCCGCCTGCAGCGCACACTGGAACGATCCCACATCCG 3’ |
|  | Qip5 | 5’ ATTACGATACGGTGCTGACC 3’ |
| ***rnhA*** | rnh-767 | 5’ CCCCCGCATGCGGATAGCCCTGCAGTGAAGCACATCAGACG 3’ |
|  | rnh-6713 | 5’ CCCCCGCATGCCGGACGAGCTCTTGTGGATCATCAAGTTGC 3’ |
|  | rnh5672 | 5’ CCCCCAGATCTGACGTCTTGCCCTTTCGCCTGGAAACG 3’ |
|  | rnh1759 | 5’ CCCCCAGATCTATGGTCAGGTGTAGCGCCCCACCC 3’ |
|  | H-up | 5’ GTCACAGAAGAGATCAATAAAAATTCTGTAGCT 3’ |
|  | H-down | 5’ TGTCAGGTGGCATTTTAGGTAGTTTGGCCTCA 3’ |
|  | sexM1750 | 5’ CCCCCGAGCTCACTCCATGGCCCAGCTCTGTGATGGTGCTGC 3’ |
| ***rdrp3*** | rdrp3clon1 | 5’ CAGCCGAGCTCAGACTAAACACGACCTTGA 3’ |
|  | rdrp3clon2 | 5’ CAACACAGAGCAAAGCAATCGC 3’ |
|  | rdrp3del1 | 5’ GAGCCGGATCCATGTCAGCCGCAGATGGAGC 3’ |
|  | rdrp3del2 | GAGGCGGATCCACAATACCGTCCCCAGTTCC |
|  | rdrp3-5new | 5’ ATGAGTGGATCAATCGTCAGG 3’ |
| ***fkbA*** | JOHE23559 | 5’ AGGAATGAGACCGGGGTAAC 3’ |
|  | JOHE23654 | 5’ **TAATACGACTCACTATAGGG**ATGGGTGTTACTGTTGAAAGAATTGCTCCT 3’ |
| ***5S rRNA*** | JOHE37682 | 5’ **TAATACGACTCACTATAGGG**AGCTACGGCCATACAATGTTG 3’ |
|  | JOHE37683 | 5’ **TAATACGACTCACTATAGGG**GAACTACAGCAACCAGTATTCCCA 3’ |
| ***P1* and *P2*** | P1 forward | 5’ AATGCCAACGAATTGAACGCCTCTTATGCT 3’ |
|  | P1 reverse | 5’ ACGAGGCATGATGACTTCGATAAAGTGC 3’ |
|  | P2 forward | 5’ AGACCGAGATTCCCAACATTGCTGCCAT 3’ |
|  | P2 reverse | 5’ CAAACTTGAGAACGCCTTGGTTTTCCAGAA 3’ |
| ***pyrG*** | pyrGZ | 5’ GGCATTGGGATGCTGTTGTC 3’ |
|  | pyrGF2 | 5’ GGCAAGTAACACCACATTCAGAGC 3’ |
|  | pyrGR2 | 5’ ATCCCACCAGAAGGAGTACATGG 3’ |
| **Others** | carB25 | 5’ CATTCCCTGTGTGGTTCTGGTTGCG 3’ |
|  | Ribo3 | 5’ CCGAATTCCTGGAGAGATGGCACCTTAGC 3’ |
|  | ago23 | 5' GCGCCCGGATCCCACTTGTCAAAAGCCCAACG 3' |
|  | ago26 | 5' CCGGCCGTCGACTATACACAAACAATCAAGGG 3' |
| ***rdrp3* cDNA analysis** | JOHE40698 | 5’ GAACGCAACGTACTGGTCGGT 3' |
|  | JOHE40699 | 5’ CGGAGTGTATACGCGCTGCT 3' |
|  | JOHE40700 | 5’ GCTTGGCGACTTCTCTGGCA 3' |
|  | JOHE40701 | 5’ GCACATGGTTCTGATAGCTGGAC 3' |
|  | JOHE40702 | 5’ CGAGAATACAACGTGGATCTGGAC 3' |
|  | JOHE40703 | 5’ TCGAGAGCACGCCGATCGCT 3' |
|  | JOHE40704 | 5’ TCCAGCTATCAGAACCATGTGCT 3' |
|  | JOHE40705 | 5’ ATGACGCCTCATCAGTCCAG 3' |
|  | JOHE40853 | 5’ AAATGTCATTATCGTCCATCTC 3' |
|  | JOHE40854 | 5’ GAGTACATTGCCGACTTCAGCAG 3' |
|  | JOHE40855 | 5’ GCAAGACATCGCTGACGCTC 3' |
| ***18S* rRNA** | S18F | 5' GGCTACCACATCCAAGGAAG 3' |
|  | S18R | 5' CTCCAATTGATCCTCGTTAA 3' |
|  |  |  |
|  |  |  |
|  |  |  |
|  |  |  |
|  |  |  |
|  |  |  |
|  |  |  |
|  |  |  |
|  |  |  |
|  |  |  |
|  |  |  |

^1^Nucleotides in red indicate restriction sites used for cloning or to release the disruption fragment from the knock-out vectors. Nucleotides in bold indicate the sequence of the T7 promotor used for in vitro transcription of RNA probes.
